# Dose–response association between moderate to vigorous physical activity and incident morbidity and mortality for individuals with a different cardiovascular health status: A cohort study among 142,493 adults from the Netherlands

**DOI:** 10.1371/journal.pmed.1003845

**Published:** 2021-12-02

**Authors:** Esmée A. Bakker, Duck-chul Lee, Maria T. E. Hopman, Eline J. Oymans, Paula M. Watson, Paul D. Thompson, Dick H. J. Thijssen, Thijs. M. H. Eijsvogels

**Affiliations:** 1 Radboud Institute for Health Sciences, Department of Physiology, Radboud University Medical Center, Nijmegen, the Netherlands; 2 Research Institute for Sports and Exercise Sciences, Liverpool John Moores University, Liverpool, United Kingdom; 3 Department of Kinesiology, Iowa State University, Ames, Iowa, United States of America; 4 Division of Cardiology, Hartford Hospital, Hartford, Connecticut, United States of America; Universite de Paris Faculte de Sante, FRANCE

## Abstract

**Background:**

Moderate to vigorous physical activity (MVPA) is strongly associated with risk reductions of noncommunicable diseases and mortality. Cardiovascular health status may influence the benefits of MVPA. We compare the association between MVPA and incident major adverse cardiovascular events (MACE) and mortality between healthy individuals, individuals with elevated levels of cardiovascular risk factors (CVRF), and cardiovascular disease (CVD).

**Methods and findings:**

A cohort study was performed in the 3 northern provinces of the Netherlands, in which data were collected between 2006 and 2018, with a median follow-up of 6.8 years (Q_25_ 5.7; Q_75_ 7.9). A total of 142,493 participants of the Lifelines Cohort Study were stratified at baseline as (1) healthy; (2) CVRF; or (3) CVD. Individuals were categorized into “inactive” and 4 quartiles of least (Q1) to most (Q4) active based on self-reported MVPA volumes. Primary outcome was a composite of incident MACE and all-cause mortality during follow-up. Cox regression was used to estimate hazard ratios (HRs), 95% confidence intervals (CIs) and *P* values. The main analyses were stratified on baseline health status and adjusted for age, sex, income, education, alcohol consumption, smoking, protein, fat and carbohydrate intake, kidney function, arrhythmias, hypothyroid, lung disease, osteoarthritis, and rheumatoid arthritis. The event rates were 2.2% in healthy individuals (*n* = 2,485 of *n* = 112,018), 7.9% in those with CVRF (*n* = 2,214 of *n* = 27,982) and 40.9% in those with CVD (*n* = 1,019 of *n* = 2,493). No linear association between MVPA and all-cause mortality or MACE was found for healthy individuals (*P* = 0.36) and individuals with CVRF (*P* = 0.86), but a linear association was demonstrated for individuals with CVD (*P* = 0.04). Adjusted HRs in healthy individuals were 0.81 (95% CI 0.64 to 1.02, *P* = 0.07), 0.71 (95% CI 0.56 to 0.89, *P* = 0.004), 0.72 (95% CI 0.57 to 0.91, *P* = 0.006), and 0.76 (95% CI 0.60 to 0.96, *P* = 0.02) for MVPA Q1 to Q4, respectively, compared to inactive individuals. In individuals with CVRF, HRs were 0.69 (95% CI 0.57 to 0.82, *P* < 0.001), 0.66 (95% CI 0.55 to 0.80, *P* < 0.001), 0.64 (95% CI 0.53 to 0.77, *P* < 0.001), and 0.69 (95% CI 0.57 to 0.84, *P* < 0.001) for MVPA Q1 to Q4, respectively, compared to inactive individuals. Finally, HRs for MVPA Q1 to Q4 compared to inactive individuals were 0.80 (95% CI 0.62 to 1.03, *P* = 0.09), 0.82 (95% CI 0.63 to 1.06, *P* = 0.13), 0.74 (95% CI 0.57 to 0.95, *P* = 0.02), and 0.70 (95% CI 0.53 to 0.93, *P* = 0.01) in CVD patients. Leisure MVPA was associated with the most health benefits, nonleisure MVPA with little health benefits, and occupational MVPA with no health benefits. Study limitations include its observational nature, self-report data about MVPA, and potentially residual confounding despite extensive adjustment for lifestyle risk factors and health-related factors.

**Conclusions:**

MVPA is beneficial for reducing adverse outcomes, but the shape of the association depends on cardiovascular health status. A curvilinear association was found in healthy and CVRF individuals with a steep risk reduction at low to moderate MVPA volumes and benefits plateauing at high(er) MVPA volumes. CVD patients demonstrated a linear association, suggesting a constant reduction of risk with higher volumes of MVPA. Therefore, individuals with CVDs should be encouraged that “more is better” regarding MVPA. These findings may help to optimize exercise prescription to gain maximal benefits of a physically active lifestyle.

## Abstract 2

Esmee Bakker and colleagues investigate the dose-response association between moderate to vigorous physical activity and incident morbidity and mortality for individuals with a different cardiovascular health status in the Netherlands.

## Author summary

**Why was this study done?:**

- There is debate to whether cardiovascular health status affects the dose–response association between physical activity (PA) and health outcomes.
- Studies among cardiovascular diseases (CVDs) patients found different associations between PA and mortality reductions, which were described as linear, J shaped, or U shaped.
- Recent studies suggested that the cardiovascular health benefits or risk of death of PA may be domain specific as different outcomes were reported for leisure versus occupational PA.

**What did the researchers do and find?:**

- A cohort study (median follow-up 6.8 years) was performed comparing the association between moderate to vigorous physical activity (MVPA) and incident major adverse cardiovascular events (MACE) and all-cause mortality between healthy individuals (*n* = 112,018), individuals with cardiovascular risk factors (CVRF) (*n* = 27,982), and CVD (*n* = 2,493).
- The shape of dose–response association between MVPA and cardiovascular events and death is curvilinear for healthy individuals and those with CVRF, whereas a linear relationship was found in individuals with CVDs.
- The association between MVPA and the risk of CVD or mortality is domain specific as leisure activities were associated with the most benefits, nonleisure activities with little benefits, and occupational activities with no benefits.

**What do these findings mean?:**

- MVPA is associated with risk reductions in all groups, but, especially, CVD patients should be encouraged that “more is better” regarding PA.
- PA recommendations could be optimized by taking cardiovascular health status and the domain of MVPA into account.

## Introduction

Regular physical activity (PA) is strongly associated with risk reductions of noncommunicable diseases and mortality [1,2]. The 2020 World Health Organization Physical Activity Guidelines recommend adults to perform at least 150 minutes/week of moderate intensity PA, or 75 minutes/week of vigorous intensity PA, or an equivalent combination of the 2. It also states that individuals with chronic diseases should not follow a “one-size-fits-all” approach and may benefit from alternative exercise prescription. This is especially relevant, given the debate as to whether health status affects the dose–response association between PA and event rate [1,3].

Data from the general population indicate that the benefits of PA on mortality and morbidity follow a curvilinear dose–response relationship [1,3–5], indicating that low or moderate volumes of PA yield a large risk reduction, whereas further increases in exercise volumes produce smaller additional benefits. By contrast, studies among cardiovascular disease (CVD) patients show conflicting results. Some studies found a linear association between PA and mortality reductions [6–8], whereas others support the presence of a reverse J-shaped or U-shaped relationship [9–12]. An important limitation of these studies is the inclusion of a single group only, with no study directly comparing the PA dose–response relationship among individuals with different cardiovascular health status.

The present study compared the association between the dose of moderate to vigorous (MV) PA and major adverse cardiovascular events (MACE) and all-cause mortality across healthy individuals, individuals with elevated levels of cardiovascular risk factors (CVRF), and individuals with CVD. We also examined the association of the specific domains of accumulating moderate to vigorous physical activity (MVPA), including leisure, nonleisure, and occupational activities on the outcomes, as recent studies suggested that the PA health benefits may differ across the domain in which PA was performed [13]. We hypothesized that the inverse curvilinear relationship between MVPA volumes and the risk of adverse outcomes, such as typically observed in the general population, will be of lower magnitude in individuals with CVD.

## Methods

### Study population

This study used prospectively gathered data from the Lifelines Cohort Study, a multidisciplinary, population-based cohort of 167,729 individuals living in the northern part of the Netherlands. Lifelines uses a broad range of procedures to assess the biomedical, sociodemographic, behavioral, physical, and psychological factors that contribute to health and disease [14,15]. All inhabitants of the northern Netherlands were eligible for Lifelines except for individuals with (1) severe psychiatric or physical illness (e.g., including individuals with cancer and associated reduced life expectancy); (2) life expectancy <5 years; and (3) lack of fluency in Dutch. Participants ≥18 years old (*n* = 152,739) were included. Participants were excluded from analyses when (1) no PA data were available (*n* = 8,666); (2) the participant had an amputated foot or leg (*n* = 165); or (3) the participant had a disease influencing their ability to be physically active, including multiple sclerosis (*n* = 347) and Parkinson disease (*n* = 76) (S1 Fig). We used the Strengthening the Reporting of Observational Studies in Epidemiology (STROBE) guideline (S1 Table) to report our findings. Participants provided written informed consent as approved by the University Medical Center Groningen Medical Ethical Committee.

### Physical examination and questionnaire

Participants received a physical examination and completed a baseline questionnaire between 2006 and 2013. The physical examination included anthropometric and blood pressure (BP) measurements. Resting systolic and diastolic BP was based on the average of 10 measurements obtained over 10 minutes using an automated sphygmomanometer (Dynamap, PRO 100V2). Blood samples were obtained after >8 hour of fasting for measurement of total, high-density lipoprotein (HDL), and low-density lipoprotein (LDL) cholesterol, triglycerides, and serum creatinine. Renal function (estimated glomerular filtration rate, eGFR) was estimated [16].

Questionnaires obtained general, lifestyle, and medical data. General information included age, sex, postal code, education level, and income. Income was estimated from Statistics Netherlands [17] using postal codes when not reported. Lifestyle factors included smoking status, alcohol consumption, nutrition intake, and hours of sleep per night. High alcohol consumption was defined as >14 drinks/week or >4 drinks/day for men and >7 drinks/week or >3 drinks/day for women [18]. Smoking status was categorized as currently, previously, and never. Dietary caloric (kcal), protein (g/day), fat (g/day), and carbohydrate (g/day)) intake were assessed using a food frequency questionnaire (FFQ) [19]. Total calories (kcal) and protein, fat, and carbohydrates intake (grams/day) were calculated from the FFQ. The medical history included medication use, presence of CVD, comorbidities, and other illnesses, including cancer, arthritis, multiple sclerosis, and Parkinson disease. Details on the physical examination and questionnaires are described elsewhere [14,15].

### Habitual PA volumes

Baseline PA was assessed using the Short Questionnaire to Assess Health-enhancing Physical Activity (SQUASH) [20]. SQUASH is divided into transportation, occupation, household, and leisure domains and asks for the duration and intensity of an individual’s typical weekly activities over the past 3 months. Weekly physical activities were converted to the average amount of metabolic equivalent of task (MET) minutes per week based on the compendium of physical activities [21]. MET minutes were calculated by multiplying the MET values of each activity by the duration. Only activities with an MV (≥ 3 MET) intensity were included, since these activities are specified in the PA guidelines [22]. Leisure MVPA contained all activities performed during leisure time. Nonleisure MVPA was defined as PA during transportation, occupation (i.e., intense work activities) and household activities. Subanalyses for occupational MVPA were performed, since previous studies suggest a potential harmful health effect of occupational PA [13]. Total and domain-specific MVPA was used to categorize individuals as inactive individuals (0 MET min/week of MVPA) and into quartiles of MVPA volumes (>0 MET min/week; Q1 to Q4).

### Health status

Participants were divided at baseline into (1) healthy; (2) CVRF; or (3) CVD. Healthy individuals had CVRF (i.e., BP, cholesterol and glucose) within the normal range and did not have known CVD. Individuals with CVRF had at least 1 of the following at baseline: (1) self-reported hypertension, hypercholesterolemia, or diabetes and used BP-lowering, cholesterol-lowering, or diabetic medications; or (2) had cholesterol levels ≥6.5 mmol/L or glucose levels >6.9 mmol/L fasting or >11.0 mmol/L nonfasting [23,24]; and (3) did not reported CVD. Individuals with CVD reported a history of heart failure, myocardial infarction, or stroke and used cardiovascular medication for these conditions at baseline. The classification of the 3 health status groups was mutually exclusive, meaning the participants were classified into one group.

### Clinical outcomes

The primary end point was a composite of overall adverse events including MACE and all-cause mortality including CVD mortality. Secondary outcomes were (1) all-cause mortality; and (2) a composite of CVD mortality and MACE. The national death and hospital registry of Statistics Netherlands were used to determine the primary and secondary outcomes. CVD mortality was based on the International Statistical Classification of Disease and Related Health Problems 10th Revision (ICD-10) [25] and included heart, essential hypertension, hypertensive renal, and cerebrovascular diseases deaths (I00 to I78) [25]. MACE was defined as ST-elevated myocardial infarct, non-ST–elevated myocardial infarction, stroke, chronic heart failure, acute heart failure, and major cardiothoracic interventions such as coronary artery bypass grafting, acute and elective percutaneous coronary intervention, and heart transplantation. MACE was classified using the diagnosis treatment codes of the insurance claims from the hospital registry of Statistics Netherlands. When hospital registry data were not available, self-reported MACE during follow-up were used instead. Self-reported MACE was assessed using follow-up questionnaires filled in after a median follow-up of 1.1, 2.1, and 3.8 years. For the date of the self-reported MACE event, we used the date at which the questionnaire was completed. Participants were followed until the first MACE event or death, whichever occurred first. Participants who did not reach the end point were censored at the end of the last assessment.

### Statistical analyses

Baseline characteristics were described for each group by cardiovascular health status. Normally distributed data were presented with mean (± standard deviation; SD), and non-normally distributed data with the median [interquartile range; Q_25_ to Q_75_]. For categories, the frequency with percentages were used to describe the data. To compare the differences between the 3 groups at baseline, 1-way independent analysis of variance (ANOVA), Kruskal–Wallis tests, and χ^2^ tests were performed.

Stratified Kaplan–Meier curves and log-rank tests were conducted to assess differences in outcomes between physically inactive and active individuals. The crude and adjusted hazard ratios (HRs) with 95% confidence intervals (CIs) were calculated using univariable and multivariable Cox proportional hazards modeling. Separate models were fitted for those with and without CVRF or CVD. Model 1 was adjusted for age (years) and sex (male/female). Model 2 was further adjusted for income (per 1,000 euros), education (low/moderate/high), alcohol consumption (low/high), smoking (pack years), protein (g/day), fat (g/day) and carbohydrate (g/day) intake, kidney function (mL/min/1.73 m^2^), arrhythmias (yes/no), hypothyroid (yes/no), lung disease (yes/no), osteoarthritis (yes/no), and rheumatoid arthritis (yes/no). Model 3 was further adjusted for factors within the causal pathway for CVD: glucose (mmol/L), total cholesterol (mmol/L), diastolic (mm Hg) and systolic BP (mm Hg), body mass index (BMI; (kg/m^2^)), and sleep (hours). Adjustment for covariates were similar for total, nonleisure, and occupational MVPA, but nonleisure MVPA was added to model 2 while examining the association between leisure MVPA and outcome. To investigate whether the dose–response relationship was moderated by cardiovascular health status, we tested interaction terms of health status (healthy, CVRF, and CVD) and the 5 MVPA categories (i.e., inactive group and quartiles of MVPA). In addition, to examine the shape of the dose–response associations between MVPA and the primary outcome, we performed restricted cubic spline regression analyses. We tested 3 (knots location at 0.10, 0.50, and 0.90 percentile), 4 (knots location at 0.05, 0.35, 0.65, and 0.95 percentile), and 5 knots (knots location at 0.05, 0.275, 0.50, 0.725, and 0.95 percentile) and calculated the Akaike information criterion to identify the best fit model [26].

Missing data of covariates were imputed with multiple imputations by chained equations with predictive mean matching [27] since 14% (*n* = 20,321) of the individuals had missing data for one of the covariates used in the model adjustments. We checked patterns of missing data and followed the “missing at random” assumption. All available variables were used to predict missing values in 5 imputed datasets with 20 iterations. Healthy convergence, imputed distribution, and plausibility were verified.

Sensitivity analyses were performed to assess the potential presence of reverse causation bias and the effect of the age difference between the 3 groups. For this purpose, we excluded participants who experienced an event and/or were censored within 2 years of follow-up, and we restricted the analyses to participants with an age above 50 years. Furthermore, effect modification (i.e., interaction terms and stratified analyses) was tested for age, sex, and education. All statistical analyses were performed in R version 3.5.2 using the following packages: survival [28], survminer [29], mice [27], and rms [30]. *P* values <0.05 were considered statistically significant.

## Results

### Study population

A total of 143,483 participants were evaluated for inclusion, of which 990 were excluded, leaving 142,493 participants available for analyses (S1 Fig). Mean age (42 years [SD 12)), proportion of males (40%), and BMI (25 [Q_25_ 23 to Q_75_ 28] were lower in the healthy individuals than in those with CVRF (54 years [SD 11], 45% male, BMI 27 [Q_25_ 25, Q_75_ 30]) or those with CVD (60 years [SD 11], 65% male, BMI 28 [Q_25_ 25, Q_75_ 31]) (Table 1). Healthy individuals were more often current smokers, had lower systolic and diastolic BP and triglycerides, higher HDL, lower serum creatinine, and fewer comorbidities. MVPA volumes were highest in healthy individuals (3,666 MET min/week [Q_25_ 1,825; Q_75_ 7,344]), followed by individuals with CVRF (3,420 MET min/week [Q_25_ 1,674; Q_75_ 6,567]) and CVD (3,333 MET min/week [Q_25_ 1,460; Q_75_ 6,093]). MVPA was mostly performed during leisure time in all 3 groups. The median percentage of MVPA spent during leisure time was 68% for the healthy, 78% for CVRF, and 89% for CVD.

**Table 1:** Baseline characteristics of the study population stratified by health status.

General characteristics	Healthy individuals *N* = 112,018	Individuals with CVRF *N* = 27,982	Individuals with CVD *N* = 2,493
Sex (male)	44,924 (40%)	12,561 (45%)	1.620 (65%)
Age (years)	42 (12)	54 (11)	60 (11)
Income × €1,000/year	28.08 (5.00)	27.97 (4.84)	27.67 (4.94)
Education level Low Moderate High	28,009 (26%) 46,221 (42%) 35,577 (32%)	11,851 (44%) 8,937 (33%) 6,282 (23%)	1,219 (51%) 671 (28%) 495 (21%)
BMI (kg/m^2^)	25 [23, 28]	27 [25, 30]	28 [25, 31]
Lifestyle characteristics			
Smoking status Never Previous Current	54,253 (49%) 32,622 (30%) 23,816 (22%)	10,394 (37%) 12,208 (44%) 5,174 (19%)	656 (26%) 1,400 (56%) 427 (17%)
Alcohol consumption Low High	80,089 (76%) 25,564 (24%)	20,269 (75%) 6,612 (25%)	1,942 (81%) 455 (19%)
PA volumes (MET minutes/week) Total Leisure Nonleisure Occupation	3,666 [1,825, 7,344] 1,953 [930, 3,531] 816 [258, 3,318] 0 [0, 1,950]	3,420 [1,674, 6,567] 2,072 [930, 3,780] 516 [22, 2,055] 0 [0, 0]	3,333 [1,460, 6,093] 2,160 [900, 4,050] 258 [0, 1,548] 0 [0, 0]
Nutrition intake Calories (kcal) Protein (g/day) Fat (g/day) Carbohydrate (g/day)	1,890 (866) 69 (29) 75 (38) 213 (102)	1,859 (751) 70 (26) 74 (34) 204 (87)	1,865 (700) 71 (25) 74 (33) 205 (83)
Medication use			
Antiplatelet	94 (0%)	83 (0%)	136 (6%)
Antihypertensive	211 (0%)	7,364 (26%)	1,130 (45%)
Anticoagulant	335 (0%)	424 (2%)	279 (11%)
Acetylsalicylic	731 (1%)	1,660 (6%)	1,621 (65%)
Anti-arrhythmic	244 (0%)	343 (1%)	113 (5%)
Beta-blocker	1,066 (1%)	5,271 (19%)	1,137 (46%)
Calcium antagonist	291 (0%)	1,956 (7%)	417 (17%)
Diuretics	213 (0%)	4,650 (17%)	499 (20%)
Statins	204 (0%)	6,126 (22%)	1,454 (58%)
Alternative cholesterol-lowering medication	6 (0%)	305 (1%)	114 (5%)
Antidiabetics	0 (0%)	2,121 (8%)	200 (8%)
Confirmed medication for CVD	0 (0%)	0 (0%)	1,881 (75.5%)
Health characteristics			
Systolic BP (mm Hg)	123 (14)	133 (16)	130 (17)
Diastolic BP (mm Hg)	73 (9)	77 (10)	74.62 (9)
Total cholesterol (mmol/L)	4.9 (0.8)	5.87 (1.28)	4.56 (1.05)
LDL cholesterol (mmol/L)	3.1 (0.8)	3.89 (1.16)	2.78 (0.95)
HDL cholesterol (mmol/L)	1.5 (0.4)	1.47 (0.43)	1.33 (0.37)
Triglycerides (mmol/L)	0.9 [0.7, 1.3]	1.3 [1.0, 1.9]	1.2 [0.9, 1.7]
Renal function (mL/min/1.73 m^2^)	99.8 [89.1, 100.0]	90.7 [79.8, 100.0]	85.5 [73.5, 95.5]
Presence of comorbidities			
Diabetes	0 (0%)	3,411 (12%)	303 (12%)
Hypertension	0 (0%)	12,300 (44%)	1,258 (51%)
Hypercholesterolemia	0 (0%)	18,004 (65%)	1,278 (51%)
Arrhythmia	6,098 (5%)	3,566 (13%)	807 (32%)
Hypothyroid	2,741 (2%)	1,307 (5%)	94 (4%)
Lung disease	13,238 (12%)	3,550 (13%)	379 (15%)
Osteoarthritis	6,159 (6%)	4,045 (15%)	432 (17%)
Rheumatoid arthritis	1,890 (2%)	946 (3%)	123 (5%)

Data are presented as mean (SD), median [Q25, Q75], and *n* (%).

BMI, body mass index; BP, blood pressure; CVD, cardiovascular disease; CVRF, cardiovascular risk factor; HDL, high-density lipoprotein; LDL, low-density lipoprotein; PA, physical activity; MET, metabolic equivalent of task.

### Clinical outcomes

**Fig 1:**
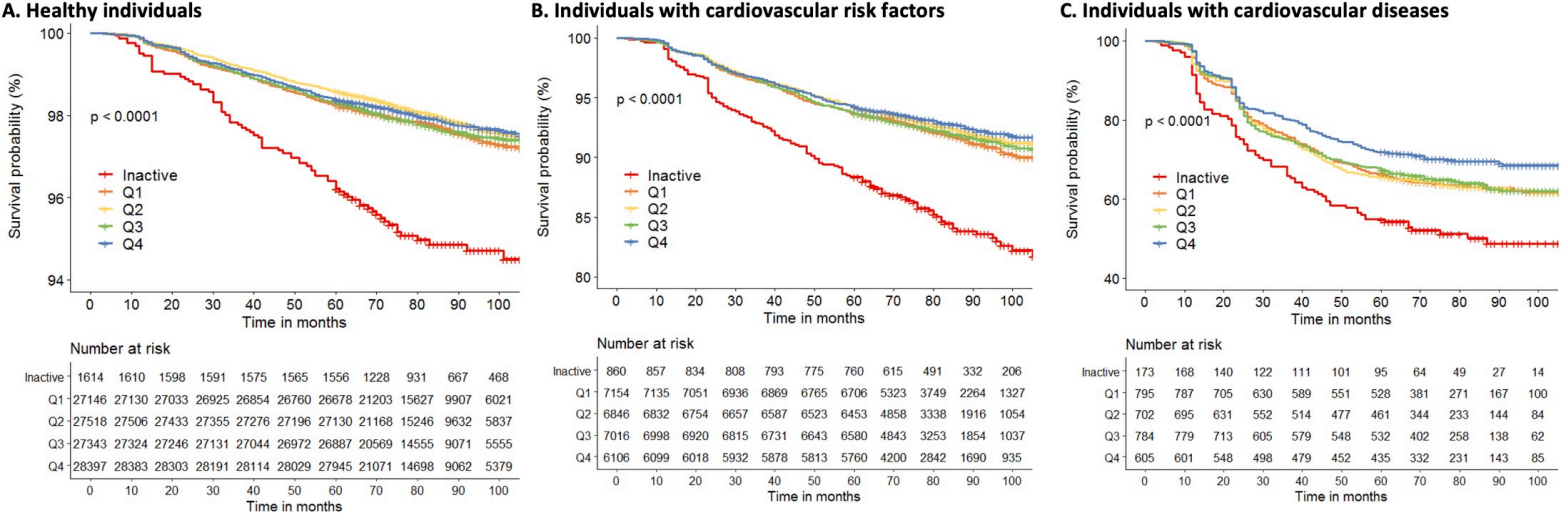
Unadjusted Kaplan–Meier estimates of all-cause mortality and MACE for quartiles of total MVPA during follow-up stratified for healthy individuals **(A)**, individuals with elevated levels of CVRF **(B)**, and individuals with CVD **(C)**. Inactive participants had a significantly lower event-free survival compared with physically active individuals (Q1 to Q4). CVD, cardiovascular disease; CVRF, cardiovascular risk factors; MACE, major adverse cardiovascular events; MVPA, moderate to vigorous physical activity.

During a median follow-up of 6.8 years (Q_25_ 5.7; Q_75_ 7.9), 5,799 participants reached the primary end point: 1,605 died and 4,194 had a MACE. The event rates were 2.2% in healthy individuals (2,485 of 112,018), 7.9% in those with CVRF (2,214 of 27,982) and 40.9% in those with CVD (1,019 of 2,493). Stratified Kaplan–Meier analyses showed a significantly higher event free survival among the active individuals compared to inactive individuals (Fig 1).

### Health benefits of MVPA

There was no linear association between MVPA and the risk of all-cause mortality and MACE for healthy individuals (HR 0.998 per 500 MET min/week [95% CI 0.993 to 1.00], *P* = 0.36) and individuals with CVRF (HR 0.997 per 500 MET min/week [95% CI 0.986 to 1.01], *P* = 0.86) (Table 2). However, the linear association between MVPA and health outcomes was significant for individuals with CVD (HR 0.991 [95%CI 0.983 to 0.999], *P* = 0.04). Based on the restricted cubic spline regression model, we found statistically significant *P* values of nonlinearity for healthy individuals and those with CVRF (*P* = 0.002 and *P* < 0.001, respectively), suggesting a nonlinear association between MVPA and MACE and mortality.

**Table 2:** HRs [95% CI] for the adverse outcomes by total MVPA.

Total PA (MET min/week)	Primary outcome—All-cause mortality and incident MACE			
	Unadjusted model	Model 1, adjusted for age and sex	Model 2, adjusted for confounders*	Model 3, adjusted for confounders and mediators^†^
**Healthy individuals**				
Continuous	0.999 [0.999; 0.999]	0.999 [0.999;1.00]	0.999 [0.999;1.00]	1.00 [0.999;1.00]
Continuous per 500 MET min/week	0.994 [0.990; 0.998]	0.998 [0.993;1.00]	0.998 [0.993;1.00]	0.998 [0.994;1.00]
*P* for linear trend	0.007	0.31	0.36	0.54
Quartiles Inactive (*n* = 1,614) Q1 1 to 1,912 (*n* = 27,146) Q2 1,913 to 3,690 (*n* = 27,518) Q3 3,690 to 7,257 (*n* = 27,343) Q4 >7,527 (*n* = 28,397)	1 0.47 [0.37; 0.59], *P* < 0.001 0.41 [0.33; 0.52], *P* < 0.001 0.46 [0.36; 0.58], *P* < 0.001 0.42 [0.34; 0.54], *P* < 0.001	1 0.68 [0.54;0.86], *P* 0.001 0.57 [0.45;0.72], *P* < 0.001 0.57 [0.45;0.72], *P* < 0.001 0.63 [0.50;0.80], *P* < 0.001	1 0.81 [0.64;1.02], *P* 0.07 0.71 [0.56;0.89], *P* 0.004 0.72 [0.57;0.91], *P* 0.006 0.76 [0.60;0.96], *P* 0.02	1 0.83 [0.66;1.05], *P* 0.13 0.73 [0.58;0.93], *P* 0.01 0.75 [0.60;0.65], *P* 0.02 0.80 [0.63;1.01], *P* 0.06
**Individuals with CVRF**				
Continuous	0.999 [0.999; 0.999]	0.999 [0.999; 1.00]	0.999 [0.999; 1.00]	1.00 [0.999; 1.00]
Continuous per 500 MET min/week	0.987 [0.978; 0.998]	0.993 [0.998; 1.00]	0.997 [0.986; 1.01]	0.998 [0.987; 1.01]
*P* for linear trend	0.004	0.44	0.86	0.73
Quartiles Inactive (*n* = 860) Q1 1 to 1,912 (*n* = 7,154) Q2 1,913 to 3,690 (*n* = 6,846) Q3 3,690 to 7,257 (*n* = 7,016) Q4 >7,527 (*n* = 6,106)	1 0.50 [0.41; 0.60], *P* < 0.001 0.46 [0.38; 0.55], *P* < 0.001 0.48 [0.40; 0.58], *P* < 0.001 0.44 [0.36; 0.53], *P* < 0.001	1 0.65 [0.54;0.79], *P* < 0.001 0.60 [0.50;0.73], *P* < 0.001 0.58 [0.48;0.70], *P* < 0.001 0.63 [0.52;0.76], *P* < 0.001	1 0.69 [0.57;0.82], *P* < 0.001 0.66 [0.55;0.80], *P* < 0.001 0.64 [0.53;0.77], *P* < 0.001 0.69 [0.57;0.84], *P* < 0.001	1 0.73 [0.60;0.87], *P* < 0.001 0.72 [0.60;0.88], *P* < 0.001 0.70 [0.58;0.85], *P* < 0.001 0.73 [0.63;0.93], *P* 0.008
**Individuals with CVD**				
Continuous	0.999 [0.999; 0.999]	0.999 [0.999; 0.999]	0.999 [0.999; 0.999]	0.999 [0.999; 0.999]
Continuous per 500 MET min/week	0.987 [0.979; 0.994]	0.990 [0.982; 0.997]	0.991 [0.983;0.999]	0.993 [0.984;1.00]
*P* for linear trend	0.001	0.01	0.04	0.08
Quartiles Inactive (*n* = 147) Q1 1 to 1,912 (*n* = 643) Q2 1,913 to 3,690 (*n* = 589) Q3 3,690 to 7,257 (*n* = 660) Q4 >7,527 (*n* = 454)	1 0.69 [0.54; 0.89], *P* 0.004 0.65 [0.51; 0.84], *P* < 0.001 0.62 [0.48; 0.84], *P* < 0.001 0.56 [0.43; 0.74], *P* < 0.001	1 0.71 [0.56; 0.91], *P* 0.008 0.68 [0.53; 0.89], *P* 0.003 0.62 [0.48; 0.80], *P* < 0.001 0.61 [0.47; 0.80], *P* < 0.001	1 0.80 [0.62; 1.03], *P* 0.09 0.82 [0.63; 1.06], *P* 0.13 0.74 [0.57; 0.95], *P* 0.02 0.70 [0.53; 0.93], *P* 0.01	1 0.79 [0.62; 1.02], *P* 0.07 0.81 [0.62; 1.05], *P* 0.11 0.74 [0.57; 0.96], *P* 0.02 0.72 [0.54; 0.94], *P* 0.02

Model 1 was adjusted for age and sex.

* Model 2 was additional adjusted for confounders: income, education, alcohol consumption, smoking behavior (pack years), nutrient intake (i.e., protein (g/day), fat (g/day), carbohydrate (g/day)), kidney function, arrhythmia, hypothyroid, lung disease, osteoarthritis, and rheumatoid arthritis.

^†^ Model 3 was further adjusted for mediators: glucose levels, total cholesterol, diastolic BP, systolic BP, BMI, and sleep.

BMI, body mass index; BP, blood pressure; CI, confidence interval; CVD, cardiovascular disease; CVRF, cardiovascular risk factor; HR, hazard ratio; MACE, major adverse cardiovascular event; MET, metabolic equivalent of task.

**Fig 2:**
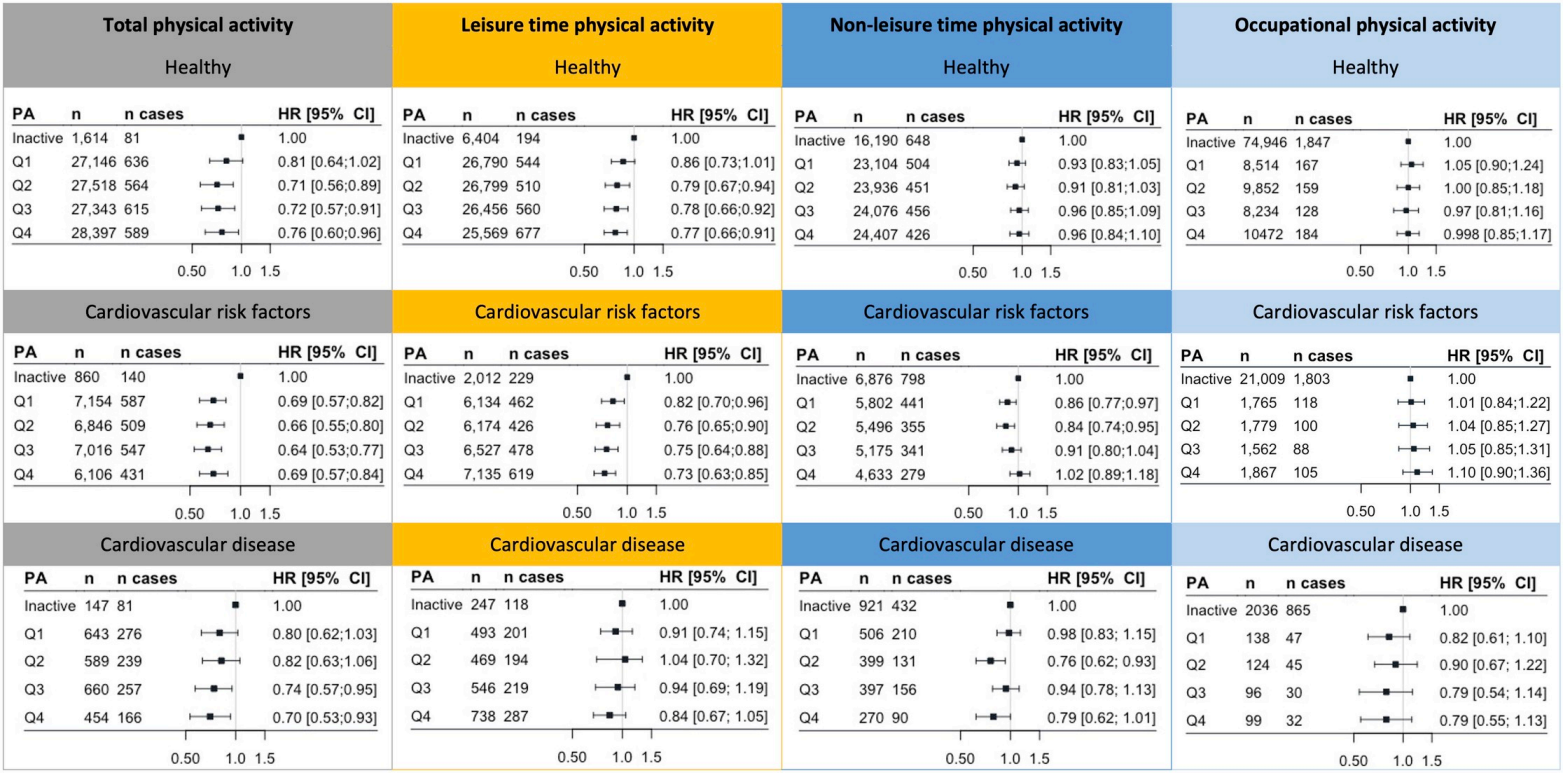
Quartiles of total and domain-specific MVPA associated with all-cause mortality and MACE stratified on health status. HRs were adjusted for age, sex, income, education, alcohol consumption, smoking behavior (pack years), nutrient intake (i.e., protein (g/day), fat (g/day), carbohydrate (g/day)), kidney function, arrhythmia, hypothyroid, lung disease, osteoarthritis, and rheumatoid arthritis. Higher levels of MVPA were associated with significant MACE and mortality risk reductions in all groups, but the effects are health status and domain dependent. CI, confidence interval; HR, hazard ratio; MACE, major adverse cardiovascular events; MVPA, moderate to vigorous physical activity; PA, physical activity.

Stratified and unadjusted analyses using quartiles of MVPA showed that active individuals showed a significantly lower risk of all-cause mortality and MACE compared to inactive individuals (Fig 2, Table 2). After adjustment for confounders (model 2), healthy individuals demonstrated significantly lower HRs within the second (HR 0.71 [95% CI 0.56 to 0.89], *P* = 0.004), third (HR 0.72 [95% CI 0.57 to 0.91], *P* = 0.006), and fourth quartile (HR 0.76 [95% CI 0.60 to 0.96], *P* = 0.02) of total MVPA, compared to inactive individuals. Participants with CVRF had significantly lower HRs within all quartiles of total MVPA (HRs Q1 0.69 [95% CI 0.57 to 0.82], *P* < 0.001, Q2 0.66 [95% CI 0.55 to 0.80], *P* < 0.001, Q3 0.64 [95% CI 0.53 to 0.77], *P* < 0.001, and Q4 0.69 [95% CI 0.57 to 0.84], *P* < 0.001). By contrast, in participants with CVD, only the third and fourth most active quartiles showed significant reductions in all-cause mortality and MACE (HRs Q1 0.80 [95% CI 0.62 to 1.03], *P* = 0.09, Q2 0.82 [95% CI 0.63 to 1.06], *P* = 0.13, Q3 0.74 [95% CI 0.57 to 0.95], *P* = 0.02, and Q4 0.70 [95% CI 0.53 to 0.93], *P* = 0.01).

Accordingly, the relationship between MVPA and health outcomes differed between healthy individuals and those with CVD (*P* for interaction: Q1 *P* = 0.39, Q2 *P* = 0.01, Q3 *P* = 0.20, and Q4 *P* = 0.11; Fig 2, Table 2). After further adjustment for additional covariates (i.e., mediators; model 3), reductions in adverse outcomes persisted with increasing MVPA, but many estimates were no longer statistically significant, especially in healthy individuals. Repeating these analyses for secondary outcomes largely reinforced the relation between MVPA and event rate (S2 and S3 Tables). Our sensitivity analyses (S4 Table) confirmed our main analyses and indicated that reverse causation bias and age restriction did not substantially change the results.

### Dose–response relationship of total MVPA

**Fig 3:**
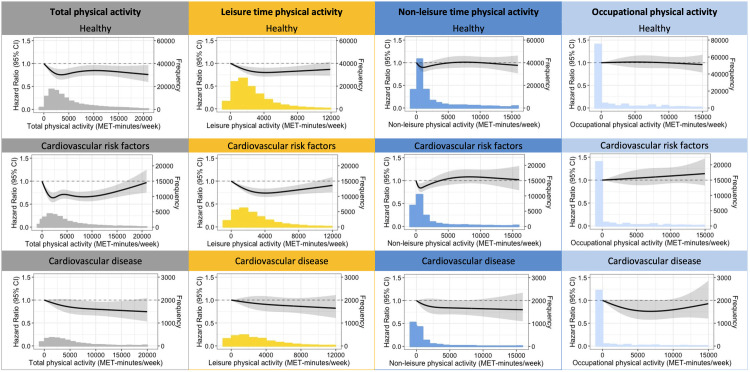
Association between total and domain-specific MVPA and all-cause mortality and MACE stratified. HRs were adjusted for age, sex, income, education, alcohol consumption, smoking behavior (pack years), nutrient intake (i.e., protein (g/day), fat (g/day), carbohydrate (g/day)), kidney function, arrhythmia, hypothyroid, lung disease, osteoarthritis, and rheumatoid arthritis. Frequency is the number of individuals within a PA category (per 1,000 MET min/week). Higher levels of MVPA were associated with significant MACE and mortality risk reductions in all groups, but the effects are health status and domain dependent. CI, confidence interval; HR, hazard ratio; MACE, major adverse cardiovascular events; MVPA, moderate to vigorous physical activity; PA, physical activity.

Increasing MVPA levels reduced the risk in all groups (Fig 3). The shape of the dose–response association was curvilinear for individuals with CVRF and healthy individuals, suggesting no additional health benefits above a certain volume of PA. This finding contrasts to CVD patients, who showed a linear dose–response association suggesting no maximal effect of PA on health. The magnitude and shape of the dose–response relationship of total MVPA and the primary outcome differed significantly between healthy individuals and those with CVD at the first part (i.e., at lower MVPA levels) of the dose–response association (*P* for interaction: spline 1 = 0.004, spline 2 = 0.04, spline 3 = 0.08, and spline 4 = 0.21). Finally, different dose–response associations were also found for education levels in healthy individuals (*P* value for interaction: spline 1 = 0.21, spline 2 = 0.061, and spline 3 = 0.048). Healthy individuals with low education had a U-shaped dose–response relationship as high MVPA volumes resulted in attenuated health benefits (S2 Fig). Age and sex did not affect the dose–response association of MVPA.

### Dose–response relationship of domain-specific MVPA

The amount of total MVPA is largely determined by leisure PA (Table 1). In line with total MVPA, participation in leisure MVPA was associated with a progressive reduction in adverse outcomes, with more leisure MVPA associated with greater reductions in all-cause mortality and incident MACE in healthy individuals and those with CVRF (Figs 2 and 3, S5–S7 Tables). However, leisure MVPA was not associated with all-cause mortality and MACE in those with CVD. Associations between nonleisure MVPA and adverse outcomes were less consistent (Figs 2 and 3, S8–S10 Tables). Risk estimates for nonleisure MVPA quartiles were <1 in healthy individuals, but did not reach statistical significance. In individuals with CVRF, nonleisure MVPA Q1 (HR 0.86 [95% CI 0.77; 0.97], *P* = 0.02) and Q2 (HR 0.84 [95% CI 0.74; 0.95], *P* = 0.006) were associated with a reduced event rate, but risk reductions were not present in nonleisure MVPA Q3 and Q4. In individuals with CVD, only nonleisure MVPA Q2 (HR 0.76 [95% CI 0.62 to 0.93], *P* = 0.008) was associated with a lower event rate compared to inactive individuals. The results for leisure or nonleisure MVPA and CVD-related mortality and MACE were comparable. The results for all-cause mortality were stronger for leisure MVPA and weaker for nonleisure MVPA (S7 and S10 Tables). When we separated occupational MVPA from nonleisure MVPA, we found no associations between occupational MVPA and the primary and secondary outcomes (Figs 2 and 3, S11–S13 Tables).

## Discussion

It is widely acknowledged that regular PA is strongly associated with a reduced risk of noncommunicable diseases and mortality [1,2], but the influence of cardiovascular health status on this association is less clear. We observed that greater amounts of total MVPA were associated with a lower risk of all-cause mortality and MACE in a curvilinear dose-dependent manner in healthy individuals and those with CVRF, with benefits plateauing at high PA volumes. By contrast, the dose–response curve of MVPA in individuals with CVD indicated a linear association. The risk reductions were primarily derived from leisure MVPA, since health benefits associated with nonleisure MVPA, including occupational MVPA, were largely insignificant. Our results suggest that the shape of the dose–response relationship between MVPA and all-cause mortality and MACE is different for individuals with CVD compared to healthy individuals and those with CVRF.

### Cardiovascular health status and MVPA benefits

The MVPA volume needed to achieve the maximal risk reduction was higher in CVD individuals (i.e., Q4; HR 0.70) compared to healthy individuals (i.e., Q2; HR 0.71) and those with CVRF (i.e., Q3; HR 0.64). These findings suggest that CVD patients need to perform more MVPA to obtain similar health benefits as healthy and CVRF individuals, despite the magnitude of the risk reduction is comparable between healthy individuals and those with CVD. Our findings reinforce observations from a large Korean study, in which PA-induced mortality reductions differed for primary and secondary prevention [8]. The potential explanation for why CVD patients have a different dose–response association for MVPA to reduce events is currently not clear, but CVD patients may require greater exercise stimuli to alter CVRF or their hemodynamic response to exercise [31] or experience an attenuated adaptations to exercise due to CVD medications [32]. Alternatively, different pathophysiological processes, altered by different amounts of MVPA, could lead to MACE and death in patients with established CVD.

Individuals with CVRF had the largest risk reductions, which suggest that those with risk factors benefit most from an active lifestyle. This may be explained by PA-induced risk factor improvements such as increased HDL cholesterol [33] and reductions in body weight, BP [34], glucose [35], triglycerides, and inflammatory markers [36], which are all known to reduce CVD events. Furthermore, the optimal PA dose for maximal risk reduction was different for the 3 subgroups: Q2 for the healthy, Q3 for those with CVRF, and Q4 for those with CVD. These findings further emphasize that PA recommendations should not follow a “one-guideline-fits-all” approach, but underline the need for precision medicine in which PA prescription may dependent, among other factors, on an individual’s cardiovascular health status.

There is debate as to whether the health benefits of PA disappear at high levels of PA [37], especially in CVD patients [38]. We found no evidence of an upper PA limit above which there is no further benefit in those with CVD, suggesting that more PA is better. By contrast, reductions in adverse outcomes were no longer statistically significant at high PA volumes in individuals with CVRF (Fig 3). This observation agrees with most [9–11,39], but not all [6,7], previous studies. A potential explanation for the upper PA limit may be due to the transient increased risk for sudden cardiac death during exercise [40] or the use of a single measure, which might induce nondifferential measurement error in regards to outcome ascertainment [41].

### Leisure versus nonleisure MVPA

Current PA guidelines [22] do not differentiate between leisure and nonleisure MVPA. A multicountry cohort study (including 168,916 participants) reported that both higher recreational (i.e., leisure) and nonrecreational (i.e., nonleisure) PA were associated with lower mortality and fewer CVD events [42]. By contrast, a meta-analysis of 17 studies (*n* = 193,696) indicated that high volumes of occupational PA, a part of nonleisure PA, were associated with detrimental health consequences in men [13]. Looking at domain-specific PA, we observed that leisure MVPA was associated with significant risk reductions in the healthy and CVRF groups, whereas no significant effect was observed in the CVD group (Figs 2 and 3). Nonleisure MVPA appeared only beneficial at low-to-moderate volumes in individuals with CVRF and CVD and did not influence health outcomes at high volumes and in healthy individuals. Occupational MVPA was not associated with health outcomes in our study. The underlying mechanisms responsible for differences in health outcomes for MVPA spent in different domains remain unclear. Some researchers suggest different physiological responses to occupational PA, such as no improvements of cardiorespiratory fitness and increases in 24-hour heart rate, BP, and inflammation [13,43]. Others argue that the detrimental association of occupational PA is biased by inadequate classification of occupational demands and incomplete adjustment for confounding factors [44]. To overcome incomplete adjustment, we have adjusted our results for differences in education level, income, and lifestyle. Taken together, our results suggest that the benefits of MVPA are domain specific, which urges future studies to further explore this topic to ultimately improve PA prescription.

### Strengths and limitations

Strengths of this study include the large population, outcome data based on national health statistics, comprehensive measurement of MVPA, and correction for important confounders. Study limitations include (1) self-reported PA data, which may overestimate PA volume; however, overreporting generally underestimates the true effect of exercise on health outcomes [45]. Furthermore, underlying health conditions (e.g., overweight, high BP, or reduced cardiorespiratory fitness) could affect the way participants experienced the intensity of the PA that they performed, which might affect the response to the questionnaire and thereby MET score of the specific PA; (2) health status was based on questionnaires, but was cross-validated using medication data ensuring that individuals with CVRF and CVD were classified correctly; (3) the study design was observational, which is prone for residual confounding; (4) occupational PA had little variance for the nonhealthy groups, which could weaken the association between occupational PA and health outcomes; and (5) when hospital registry data were not available, self-reported MACE were used instead. The date of self-reported MACE was based on the date at which the participant filled in the follow-up questionnaire, which may slightly deviate from the date at which the MACE occurred. Since we adjusted for the most common confounding factors, we reduced the risks of residual confounding as much as possible. Finally, light intensity PA was not assessed. Previous studies using device-based measures of PA showed that light intensity PA was associated with mortality [46], suggesting that performing some PA is better than performing none [22]. Especially in individuals with CVD, starting with light intensity PA is a feasible way to increase the level of habitual PA.

## Conclusions

Higher levels of MVPA were associated with significant CVD and mortality risk reductions in all groups, but benefits plateaued at high PA volumes in healthy individuals and those with CVRF. Cardiovascular health status affected the dose–response association between PA and MACE and all-cause mortality. Specifically, in individuals with CVD, larger volumes of MVPA were linked to health benefits compared to healthy individuals and those with CVRF. Healthy controls and individuals with CVRF demonstrated a curvilinear relationship, whereas a linear association was found among individuals with CVD. Furthermore, we observed important differences in risk reductions across different domains of MVPA. Leisure MVPA was associated with the most health benefits, nonleisure MVPA with little health benefits, and occupational MVPA with no health benefits. Outcomes from this study are useful to further optimize PA recommendations, so that every individual, irrespective of cardiovascular health status, can optimally benefit from a physically active lifestyle.

## Supporting information

S1 Fig Flowchart of study population. (TIF)

S2 Fig The association between total MVPA and all-cause mortality or MACE in healthy individuals stratified for education level. HRs were adjusted for confounders (Model 2): age, sex, income, education, alcohol consumption, smoking behavior (pack years), nutrient intake (i.e., protein (g/day), fat (g/day), carbohydrate (g/day)), kidney function, arrhythmia, hypothyroid, lung disease, osteoarthritis, and rheumatoid arthritis. CI, confidence interval; HR, hazard ratio; MACE, major adverse cardiovascular events; MVPA, moderate to vigorous physical activity.
(TIFF)

S1 Table STROBE checklist. STROBE, Strengthening the Reporting of Observational Studies in Epidemiology.
(DOCX)

S2 Table HRs (95% CI) for the association between total MVPA and cardiovascular mortality and incident MACE. CI, confidence interval; HR, hazard ratio; MACE, major adverse cardiovascular events; MVPA, moderate to vigorous physical activity.
(DOCX)

S3 Table HRs (95% CI) for the association between total MVPA and all-cause mortality. CI, confidence interval; HR, hazard ratio; MVPA, moderate to vigorous physical activity.
(DOCX)

S4 Table Sensitivity analyses for reverse causation bias and age-restricted (>50 years) analyses. (DOCX)

S5 Table HRs (95% CI) for the association between leisure MVPA and all-cause mortality and MACE. CI, confidence interval; HR, hazard ratio; MACE, major adverse cardiovascular events; MVPA, moderate to vigorous physical activity.
(DOCX)

S6 Table HRs (95% CI) for the association between leisure MVPA and cardiovascular mortality and incident MACE. CI, confidence interval; HR, hazard ratio; MACE, major adverse cardiovascular events; MVPA, moderate to vigorous physical activity.
(DOCX)

S7 Table HRs (95% CI) for the association between leisure MVPA and all-cause mortality. CI, confidence interval; HR, hazard ratio; MVPA, moderate to vigorous physical activity.
(DOCX)

S8 Table HRs (95% CI) for the association between nonleisure MVPA and all-cause mortality and MACE. CI, confidence interval; HR, hazard ratio; MACE, major adverse cardiovascular events; MVPA, moderate to vigorous physical activity.
(DOCX)

S9 Table HRs (95% CI) for the association between nonleisure MVPA and cardiovascular mortality and incident MACE. CI, confidence interval; HR, hazard ratio; MACE, major adverse cardiovascular events; MVPA, moderate to vigorous physical activity.
(DOCX)

S10 Table HRs (95% CI) for the association between nonleisure MVPA and all-cause mortality. CI, confidence interval; HR, hazard ratio; MVPA, moderate to vigorous physical activity.
(DOCX)

S11 Table HRs (95% CI) for the association between occupational MVPA and all-cause mortality and incident MACE. CI, confidence interval; HR, hazard ratio; MACE, major adverse cardiovascular events; MVPA, moderate to vigorous physical activity.
(DOCX)

S12 Table HRs (95% CI) for the association between occupational MVPA and cardiovascular mortality and incident MACE. CI, confidence interval; HR, hazard ratio; MACE, major adverse cardiovascular events; MVPA, moderate to vigorous physical activity.
(DOCX)

S13 Table HRs (95% CI) for the association between occupational MVPA and all-cause mortality. CI, confidence interval; HR, hazard ratio; MVPA, moderate to vigorous physical activity.
(DOCX)

## Abbreviations

ANOVA: analysis of variance
BMI: body mass index
BP: blood pressure
CI: confidence interval
CVD: cardiovascular disease
CVRF: cardiovascular risk factors
eGFR: estimated glomerular filtration rate
FFQ: food frequency questionnaire
HDL: high-density lipoprotein
HR: hazard ratio
ICD-10: International Statistical Classification of Disease and Related Health Problems 10th Revision
LDL: low-density lipoprotein
MACE: major adverse cardiovascular events
MET: metabolic equivalent of task
MV: moderate to vigorous
MVPA: moderate to vigorous physical activity
PA: physical activity
SQUASH: Short Questionnaire to Assess Health-enhancing Physical Activity
STROBE: Strengthening the Reporting of Observational Studies in Epidemiology

## References

[1] Eijsvogels TM, Molossi S, Lee DC, Emery MS, Thompson PD. Exercise at the Extremes: The Amount of Exercise to Reduce Cardiovascular Events. J Am Coll Cardiol. 2016;67(3):316–29. Epub 2016/01/23. doi: 10.1016/j.jacc.2015.11.034 . 26796398

[2] Lee IM, Shiroma EJ, Lobelo F, Puska P, Blair SN, Katzmarzyk PT, et al. Effect of physical inactivity on major non-communicable diseases worldwide: an analysis of burden of disease and life expectancy. Lancet. 2012;380(9838):219–29. Epub 2012/07/24. doi: 10.1016/S0140-6736(12)61031-9 ; PubMed Central PMCID: PMC3645500. 22818936 PMC3645500

[3] Moore SC, Patel AV, Matthews CE, Berrington de Gonzalez A, Park Y, Katki HA, et al. Leisure time physical activity of moderate to vigorous intensity and mortality: a large pooled cohort analysis. PLoS Med. 2012;9(11):e1001335. Epub 2012/11/10. doi: 10.1371/journal.pmed.1001335 ; PubMed Central PMCID: PMC3491006. 23139642 PMC3491006

[4] Kraus WE, Powell KE, Haskell WL, Janz KF, Campbell WW, Jakicic JM, et al. Physical Activity, All-Cause and Cardiovascular Mortality, and Cardiovascular Disease. Med Sci Sports Exerc. 2019;51(6):1270–81. Epub 2019/05/17. doi: 10.1249/MSS.0000000000001939 ; PubMed Central PMCID: PMC6527136. 31095084 PMC6527136

[5] Arem H, Moore SC, Patel A, Hartge P, Berrington de Gonzalez A, Visvanathan K, et al. Leisure time physical activity and mortality: a detailed pooled analysis of the dose-response relationship. JAMA Intern Med. 2015;175(6):959–67. Epub 2015/04/07. doi: 10.1001/jamainternmed.2015.0533 ; PubMed Central PMCID: PMC4451435. 25844730 PMC4451435

[6] Moholdt T, Wisloff U, Nilsen TI, Slordahl SA. Physical activity and mortality in men and women with coronary heart disease: a prospective population-based cohort study in Norway (the HUNT study). Eur J Cardiovasc Prev Rehabil. 2008;15(6):639–45. Epub 2008/09/10. doi: 10.1097/HJR.0b013e3283101671 . 18779734

[7] Stewart RAH, Held C, Hadziosmanovic N, Armstrong PW, Cannon CP, Granger CB, et al. Physical Activity and Mortality in Patients With Stable Coronary Heart Disease. J Am Coll Cardiol. 2017;70(14):1689–700. Epub 2017/09/30. doi: 10.1016/j.jacc.2017.08.017 . 28958324

[8] Jeong SW, Kim SH, Kang SH, Kim HJ, Yoon CH, Youn TJ, et al. Mortality reduction with physical activity in patients with and without cardiovascular disease. Eur Heart J. 2019;40(43):3547–55. Epub 2019/09/11. doi: 10.1093/eurheartj/ehz564 ; PubMed Central PMCID: PMC6855138. 31504416 PMC6855138

[9] Mons U, Hahmann H, Brenner H. A reverse J-shaped association of leisure time physical activity with prognosis in patients with stable coronary heart disease: evidence from a large cohort with repeated measurements. Heart. 2014;100(13):1043–9. Epub 2014/05/16. doi: 10.1136/heartjnl-2013-305242 . 24829374

[10] Wannamethee SG, Shaper AG, Walker M. Physical activity and mortality in older men with diagnosed coronary heart disease. Circulation. 2000;102(12):1358–63. Epub 2000/09/20. doi: 10.1161/01.cir.102.12.1358 . 10993852

[11] Williams PT, Thompson PD. Increased cardiovascular disease mortality associated with excessive exercise in heart attack survivors. Mayo Clin Proc. 2014;89(9):1187–94. Epub 2014/08/17. doi: 10.1016/j.mayocp.2014.05.006 . 25128072

[12] Keteyian SJ, Leifer ES, Houston-Miller N, Kraus WE, Brawner CA, O’Connor CM, et al. Relation between volume of exercise and clinical outcomes in patients with heart failure. J Am Coll Cardiol. 2012;60(19):1899–05. Epub 2012/10/16. doi: 10.1016/j.jacc.2012.08.958 ; PubMed Central PMCID: PMC3804919. 23062530 PMC3804919

[13] Coenen P, Huysmans MA, Holtermann A, Krause N, van Mechelen W, Straker LM, et al. Do highly physically active workers die early? A systematic review with meta-analysis of data from 193 696 participants. Br J Sports Med. 2018;52(20):1320–6. Epub 2018/05/16. doi: 10.1136/bjsports-2017-098540 . 29760168

[14] Scholtens S, Smidt N, Swertz MA, Bakker SJ, Dotinga A, Vonk JM, et al. Cohort Profile: LifeLines, a three-generation cohort study and biobank. Int J Epidemiol. 2015;44(4):1172–80. Epub 2014/12/17. doi: 10.1093/ije/dyu229 . 25502107

[15] Stolk RP, Rosmalen JG, Postma DS, de Boer RA, Navis G, Slaets JP, et al. Universal risk factors for multifactorial diseases: LifeLines: a three-generation population-based study. Eur J Epidemiol. 2008;23(1):67–74. Epub 2007/12/14. doi: 10.1007/s10654-007-9204-4 . 18075776

[16] National Kidney F. K/DOQI clinical practice guidelines for chronic kidney disease: evaluation, classification, and stratification. Am J Kidney Dis. 2002;39(2 Suppl 1):S1–266. Epub 2002/03/21. . 11904577

[17] Statistics Netherlands 2020 [19–11–2020]. Available from: https://www.cbs.nl/en-gb.

[18] Fleming MF. Screening and Brief Intervention in Primary Care Settings. National institute on alcohols abuse and alcoholism. [27–1–2020]. Available from: https://pubs.niaaa.nih.gov/publications/arh28-2/57-62.htm. 19006992 PMC6601648

[19] Siebelink E, Geelen A, de Vries JH. Self-reported energy intake by FFQ compared with actual energy intake to maintain body weight in 516 adults. Br J Nutr. 2011;106(2):274–81. Epub 2011/02/23. doi: 10.1017/S0007114511000067 . 21338536

[20] Wendel-Vos GC, Schuit AJ, Saris WH, Kromhout D. Reproducibility and relative validity of the short questionnaire to assess health-enhancing physical activity. J Clin Epidemiol. 2003;56(12):1163–9. Epub 2003/12/19. doi: 10.1016/s0895-4356(03)00220-8 . 14680666

[21] Ainsworth BE, Haskell WL, Herrmann SD, Meckes N, Bassett DR Jr., Tudor-Locke C, et al. 2011 Compendium of Physical Activities: a second update of codes and MET values. Med Sci Sports Exerc. 2011;43(8):1575–81. Epub 2011/06/18. doi: 10.1249/MSS.0b013e31821ece12 . 21681120

[22] World Health Organization. WHO guidelines on physical activity and sedentary behaviour. Geneva: WHO Press; 2020. 33369898

[23] National Cholesterol Education Program Expert Panel on Detection E, Treatment of High Blood Cholesterol in A. Third Report of the National Cholesterol Education Program (NCEP) Expert Panel on Detection, Evaluation, and Treatment of High Blood Cholesterol in Adults (Adult Treatment Panel III) final report. Circulation. 2002;106(25):3143–421. Epub 2002/12/18. . 12485966

[24] American Diabetes A. 2. Classification and Diagnosis of Diabetes: Standards of Medical Care in Diabetes-2018. Diabetes Care. 2018;41(Suppl 1):S13–S27. Epub 2017/12/10. doi: 10.2337/dc18-S002 . 29222373

[25] World Health Organisation. International Statistical Classification of Diseases and Related Health Problems 10th Revision. Geneva: 2010.

[26] Durrleman S, Simon R. Flexible regression models with cubic splines. Stat Med. 1989;8(5):551–61. Epub 1989/05/01. doi: 10.1002/sim.4780080504 2657958

[27] van Buuren SG-O, K.;. MICE: Multivariate Imputation by Chained Equations in R. Journal of Statistical Software. 2011;45(3):1–67.

[28] Therneau T.A Package for Survival Analysis in S. version 2.38 2015 [1–7–2020]. Available from: https://CRAN.R-project.org/package=survival.

[29] Kassambara AK, Biecek M, Fabian P, Drawing S. Survival Curves using ’ggplot2’. 2019.

[30] Harrell FE. Package ‘rms’. Version: 5.1–4 2019 [1–7–2020]. Available from: https://cran.r-project.org/web/packages/rms/rms.pdf.

[31] Green DJ, Hopman MT, Padilla J, Laughlin MH, Thijssen DH. Vascular Adaptation to Exercise in Humans: Role of Hemodynamic Stimuli. Physiol Rev. 2017;97(2):495–528. Epub 2017/02/06. doi: 10.1152/physrev.00014.2016 ; PubMed Central PMCID: PMC5539408. 28151424 PMC5539408

[32] Mikus CR, Boyle LJ, Borengasser SJ, Oberlin DJ, Naples SP, Fletcher J, et al. Simvastatin impairs exercise training adaptations. J Am Coll Cardiol. 2013;62(8):709–14. Epub 2013/04/16. doi: 10.1016/j.jacc.2013.02.074 ; PubMed Central PMCID: PMC3745788. 23583255 PMC3745788

[33] Mann S, Beedie C, Jimenez A. Differential effects of aerobic exercise, resistance training and combined exercise modalities on cholesterol and the lipid profile: review, synthesis and recommendations. Sports Med. 2014;44(2):211–21. Epub 2013/11/01. doi: 10.1007/s40279-013-0110-5 ; PubMed Central PMCID: PMC3906547. 24174305 PMC3906547

[34] Whelton SP, Chin A, Xin X, He J. Effect of aerobic exercise on blood pressure: a meta-analysis of randomized, controlled trials. Ann Intern Med. 2002;136(7):493–503. Epub 2002/04/03. doi: 10.7326/0003-4819-136-7-200204020-00006 . 11926784

[35] Thomas DE, Elliott EJ, Naughton GA. Exercise for type 2 diabetes mellitus. Cochrane Database Syst Rev. 2006;(3):CD002968. Epub 2006/07/21. doi: 10.1002/14651858.CD002968.pub2 16855995 PMC8989410

[36] Szostak J, Laurant P. The forgotten face of regular physical exercise: a ’natural’ anti-atherogenic activity. Clin Sci (Lond). 2011;121(3):91–106. Epub 2011/07/07. doi: 10.1042/CS20100520 . 21729002

[37] Eijsvogels TMH, Thompson PD, Franklin BA. The "Extreme Exercise Hypothesis": Recent Findings and Cardiovascular Health Implications. Curr Treat Options Cardiovasc Med. 2018;20(10):84. Epub 2018/08/30. doi: 10.1007/s11936-018-0674-3 ; PubMed Central PMCID: PMC6132728. 30155804 PMC6132728

[38] Eijsvogels TMH, Thompson PD. Exercise Is Medicine At Any Dose? Jama-Journal of the American Medical Association. 2015;314(18):1915–6. WOS:000364490700010. doi: 10.1001/jama.2015.10858 26547459

[39] Armstrong ME, Green J, Reeves GK, Beral V, Cairns BJ, Million Women Study C. Frequent physical activity may not reduce vascular disease risk as much as moderate activity: large prospective study of women in the United Kingdom. Circulation. 2015;131(8):721–9. doi: 10.1161/CIRCULATIONAHA.114.010296 . 25688148

[40] Franklin BA, Thompson PD, Al-Zaiti SS, Albert CM, Hivert MF, Levine BD, et al. Exercise-Related Acute Cardiovascular Events and Potential Deleterious Adaptations Following Long-Term Exercise Training: Placing the Risks Into Perspective-An Update: A Scientific Statement From the American Heart Association. Circulation. 2020;141(13):e705–e36. Epub 2020/02/27. doi: 10.1161/CIR.0000000000000749 . 32100573

[41] Lee DH, Rezende LFM, Ferrari G, Aune D, Keum N, Tabung FK, et al. Physical activity and all-cause and cause-specific mortality: assessing the impact of reverse causation and measurement error in two large prospective cohorts. Eur J Epidemiol. 2021;36(3):275–85. Epub 2021/01/12. doi: 10.1007/s10654-020-00707-3 ; PubMed Central PMCID: PMC8035269. 33428024 PMC8035269

[42] Lear SA, Hu W, Rangarajan S, Gasevic D, Leong D, Iqbal R, et al. The effect of physical activity on mortality and cardiovascular disease in 130 000 people from 17 high-income, middle-income, and low-income countries: the PURE study. Lancet. 2017;390(10113):2643–54. Epub 2017/09/26. doi: 10.1016/S0140-6736(17)31634-3 . 28943267

[43] Holtermann A, Krause N, van der Beek AJ, Straker L. The physical activity paradox: six reasons why occupational physical activity (OPA) does not confer the cardiovascular health benefits that leisure time physical activity does. Br J Sports Med. 2018;52(3):149–50. Epub 2017/08/12. doi: 10.1136/bjsports-2017-097965 . 28798040

[44] Shephard RJ. Is there a ’recent occupational paradox’ where highly active physically active workers die early? Or are there failures in some study methods? Br J Sports Med. 2019;53(24):1557–9. Epub 2019/03/25. doi: 10.1136/bjsports-2018-100344 . 30902817

[45] Celis-Morales CA, Perez-Bravo F, Ibanez L, Salas C, Bailey ME, Gill JM. Objective vs. self-reported physical activity and sedentary time: effects of measurement method on relationships with risk biomarkers. PLoS ONE. 2012;7(5):e36345. Epub 2012/05/17. doi: 10.1371/journal.pone.0036345 ; PubMed Central PMCID: PMC3348936. 22590532 PMC3348936

[46] Ekelund U, Tarp J, Steene-Johannessen J, Hansen BH, Jefferis B, Fagerland MW, et al. Dose-response associations between accelerometry measured physical activity and sedentary time and all cause mortality: systematic review and harmonised meta-analysis. BMJ. 2019;366:l4570. Epub 2019/08/23. doi: 10.1136/bmj.l4570 ; PubMed Central PMCID: PMC6699591. 31434697 PMC6699591

