# Supplementary material for: Dose–response association between moderate to vigorous physical activity and incident morbidity and mortality for individuals with a different cardiovascular health status: A cohort study among 142,493 adults from the Netherlands
Source: PLoS Med. 2021 Dec 2;18(12):e1003845. doi: 10.1371/journal.pmed.1003845 (PMC8638933; doi:10.1371/journal.pmed.1003845)
Supplement: S1 Table — STROBE, Strengthening the Reporting of Observational Studies in Epidemiology. (DOCX) [file pmed.1003845.s003.docx]

**S1 Table.** STROBE checklist

|  | | **Item No** | **Recommendation** | **Page No** |
| --- | --- | --- | --- | --- |
| **Title and abstract** | | 1 | (*a*) Indicate the study’s design with a commonly used term in the title or the abstract | Title and abstract paragraph 2 |
|  |  |  | (*b*) Provide in the abstract an informative and balanced summary of what was done and what was found | abstract |
| **Introduction** | | | | |
| Background/rationale | | 2 | Explain the scientific background and rationale for the investigation being reported | Introduction, paragraph 1-3 |
| Objectives | | 3 | State specific objectives, including any prespecified hypotheses | Introduction, paragraph 3 |
| **Methods** | | | | |
| Study design | | 4 | Present key elements of study design early in the paper | Study population, paragraph 1 |
| Setting | | 5 | Describe the setting, locations, and relevant dates, including periods of recruitment, exposure, follow-up, and data collection | Study population , paragraph 1 |
| Participants | | 6 | (*a*) Give the eligibility criteria, and the sources and methods of selection of participants. Describe methods of follow-up | Study population, paragraph 1 and Clinical outcomes, paragraph 1 |
|  |  |  | (*b*) For matched studies, give matching criteria and number of exposed and unexposed | - |
| Variables | | 7 | Clearly define all outcomes, exposures, predictors, potential confounders, and effect modifiers. Give diagnostic criteria, if applicable | Methods, all paragraphs |
| Data sources/ measurement | | 8* | For each variable of interest, give sources of data and details of methods of assessment (measurement). Describe comparability of assessment methods if there is more than one group | Methods, all paragraphs |
| Bias | | 9 | Describe any efforts to address potential sources of bias | Statistical analyses, all paragraphs |
| Study size | | 10 | Explain how the study size was arrived at | Study population, paragraph 1 |
| Quantitative variables | | 11 | Explain how quantitative variables were handled in the analyses. If applicable, describe which groupings were chosen and why | Statistical analyses, paragraph 2 |
| Statistical methods | | 12 | (*a*) Describe all statistical methods, including those used to control for confounding | Statistical analyses, all paragraphs |
|  |  |  | (*b*) Describe any methods used to examine subgroups and interactions | Statistical analyses, paragraph 2 to 4 |
|  |  |  | (*c*) Explain how missing data were addressed | Statistical analyses, paragraph 3 |
|  |  |  | (*d*) If applicable, explain how loss to follow-up was addressed | Clinical outcomes, paragraph 1 |
|  |  |  | (*e*) Describe any sensitivity analyses | Statistical analyses, paragraph 4 |
| **Results** | | | |  |
| Participants | | 13* | (a) Report numbers of individuals at each stage of study—eg numbers potentially eligible, examined for eligibility, confirmed eligible, included in the study, completing follow-up, and analysed | Study population (methods), paragraph 1 and Study population (results), paragraph 1 |
|  |  |  | (b) Give reasons for non-participation at each stage | Study population (methods), paragraph 1 and Study population (results), paragraph 1 and |
|  |  |  | (c) Consider use of a flow diagram | S1 fig |
| Descriptive data | | 14* | (a) Give characteristics of study participants (eg demographic, clinical, social) and information on exposures and potential confounders | Study population (results), paragraph 1 and table 1 |
|  |  |  | (b) Indicate number of participants with missing data for each variable of interest | - |
|  |  |  | (c) Summarise follow-up time (eg, average and total amount) | Clinical outcomes, paragraph 1 |
| Outcome data | | 15* | Report numbers of outcome events or summary measures over time | Clinical outcomes, paragraph 1 |
| Main results | 16 | (*a*) Give unadjusted estimates and, if applicable, confounder-adjusted estimates and their precision (eg, 95% confidence interval). Make clear which confounders were adjusted for and why they were included | | Health benefits of MVPA to Dose-response relationship of domain-specific MVPA and Tables 2, S2 t/m S12 |
|  |  | (*b*) Report category boundaries when continuous variables were categorized | | Statistical analyses paragraph 2 |
|  |  | (*c*) If relevant, consider translating estimates of relative risk into absolute risk for a meaningful time period | | - |
| Other analyses | 17 | Report other analyses done—eg analyses of subgroups and interactions, and sensitivity analyses | | Health benefits of MVPA, paragraphs 2 and 3 |
| **Discussion** | | | | |
| Key results | 18 | Summarise key results with reference to study objectives | | Discussion, paragraph 1 |
| Limitations | 19 | Discuss limitations of the study, taking into account sources of potential bias or imprecision. Discuss both direction and magnitude of any potential bias | | Strengths and limitations, paragraph 1 |
| Interpretation | 20 | Give a cautious overall interpretation of results considering objectives, limitations, multiplicity of analyses, results from similar studies, and other relevant evidence | | Cardiovascular health status and MVPA benefits to Leisure versus non-leisure MVPA |
| Generalisability | 21 | Discuss the generalisability (external validity) of the study results | | Cardiovascular health status and MVPA benefits to Strengths and limitations |
| **Other information** | | | | |
| Funding | 22 | Give the source of funding and the role of the funders for the present study and, if applicable, for the original study on which the present article is based | | Title page, funding/support |

*Give information separately for exposed and unexposed groups.
