# Supplementary material for: Dose–response association between moderate to vigorous physical activity and incident morbidity and mortality for individuals with a different cardiovascular health status: A cohort study among 142,493 adults from the Netherlands
Source: PLoS Med. 2021 Dec 2;18(12):e1003845. doi: 10.1371/journal.pmed.1003845 (PMC8638933; doi:10.1371/journal.pmed.1003845)
Supplement: S2 Table — CI, confidence interval; HR, hazard ratio; MACE, major adverse cardiovascular events; MVPA, moderate to vigorous physical activity. (DOCX) [file pmed.1003845.s004.docx]

| **S2 Table.** Hazard ratios (95% CI) for the association between total moderate to vigorous physical activity and cardiovascular mortality and incident MACE. | | | | |
| --- | --- | --- | --- | --- |
| **Total physical activity (MET-min/week)** | **Secondary outcome – Cardiovascular mortality and incident MACE** | | | |
|  | Unadjusted model | Model 1, adjusted for age and sex | Model 2, adjusted for confounders* | Model 3, adjusted for confounders and mediators† |
| **Healthy individuals** | | |  |  |
| Continuous | 0.999 [0.999; 0.999] | 0.999 [0.999;1.00] | 0.999 [0.999;1.00] | 1.00 [0.999;1.00] |
| P for linear trend | 0.04 | 0.27 | 0.27 | 0.39 |
| Quartiles  Inactive  Q1 1-1912  Q2 1913-3690  Q3 3690-7257  Q4 >7527 | 1  0.48 [0.36; 0.63]  0.40 [0.30; 0.53]  0.45 [0.34; 0.60]  0.43 [0.34; 0.57] | 1  0.69 [0.52;0.92]  0.55 [0.41;0.73]  0.56 [0.42;0.74]  0.63 [0.47;0.83] | 1  0.84 [0.64;1.12]  0.70 [0.53;0.94]  0.72 [0.54;0.96]  0.77 [0.58;1.03] | 1  0.87 [0.65;1.15]  0.73 [0.55;0.97]  0.76 [0.57;1.01]  0.81 [0.61;1.08] |
| **Individuals with CVRF** | | |  |  |
| Continuous | 0.999 [0.999; 1.00] | 1.00 [0.999;1.00] | 1.00 [0.999;1.00] | 1.00 [0.999;1.00] |
| P for linear trend | 0.07 | 0.92 | 0.57 | 0.29 |
| Quartiles  Inactive  Q1 1-1912  Q2 1913-3690  Q3 3690-7257  Q4 >7527 | 1  0.51 [0.41; 0.63]  0.48 [0.39; 0.60]  0.52 [0.42; 0.65]  0.48 [0.39; 0.60] | 1  0.65 [0.53;0.81]  0.62 [0.50;0.77]  0.60 [0.49;0.75]  0.66 [0.53;0.82] | 1  0.69 [0.56;0.86]  0.69 [0.55;0.86]  0.68 [0.54;0.84]  0.73 [0.58;0.91] | 1  0.73 [0.59;0.91]  0.74 [0.60;0.93]  0.73 [0.59;0.91]  0.79 [0.63;0.996] |
| **Individuals with CVD** | | |  |  |
| Continuous | 0.999 [0.999; 0.999] | 0.999 [0.999;0.999] | 0.999 [0.999; 0.999] | 0.999 [0.999; 0.999] |
| P for linear trend | 0.001 | 0.009 | 0.02 | 0.04 |
| Quartiles  Inactive  Q1 1-1912  Q2 1913-3690  Q3 3690-7257  Q4 >7527 | 1  0.70 [0.54; 0.91]  0.67 [0.51; 0.87]  0.63 [0.48; 0.82]  0.56 [0.42; 0.74] | 1  0.71 [0.56; 0.93]  0.70 [0.53; 0.91]  0.64 [0.49; 0.83]  0.61 [0.47; 0.80] | 1  0.81 [0.62; 1.06]  0.84 [0.64; 1.10]  0.76 [0.58; 0.99]  0.69 [0.52; 0.92] | 1  0.80 [0.61; 1.04]  0.83 [0.63; 1.09]  0.76 [0.58; 0.997]  0.70 [0.53; 0.94] |
| Model 1 was adjusted for age and sex. *Model 2 was additional adjusted for confounders: income, education, alcohol consumption, smoking behaviour (packyears), nutrient intake (i.e. protein (g/day), fat (g/day), carbohydrate (g/day)), kidney function, arrhythmia, hypothyroid, lung disease, osteoarthritis and rheumatoid arthritis. †Model 3 is further adjusted for mediators: glucose levels, total cholesterol, diastolic blood pressure, systolic blood pressure, BMI, and sleep. | | | | |
