# Supplementary material for: Dose–response association between moderate to vigorous physical activity and incident morbidity and mortality for individuals with a different cardiovascular health status: A cohort study among 142,493 adults from the Netherlands
Source: PLoS Med. 2021 Dec 2;18(12):e1003845. doi: 10.1371/journal.pmed.1003845 (PMC8638933; doi:10.1371/journal.pmed.1003845)
Supplement: S3 Table — CI, confidence interval; HR, hazard ratio; MVPA, moderate to vigorous physical activity. (DOCX) [file pmed.1003845.s005.docx]

| **S3 Table.** Hazard ratios (95% CI) for the association between total moderate to vigorous physical activity and all-cause mortality . | | | | |
| --- | --- | --- | --- | --- |
| **Total physical activity (MET-min/week)** | **Secondary outcome – All-cause mortality** | | | |
|  | Unadjusted model | Model 1, adjusted for age and sex | Model 2, adjusted for confounders* | Model 3, adjusted  for confounders  and mediators† |
| **Healthy individuals** |  |  |  |  |
| Continuous | 0.999 [0.999; 0.999] | 0.999 [0.999;1.00] | 0.999 [0.999;1.00] | 0.999 [0.999;1.00] |
| P for linear trend | 0.006 | 0.27 | 0.42 | 0.55 |
| Quartiles  Inactive  Q1 1-1912  Q2 1913-3690  Q3 3690-7257  Q4 >7527 | 1  0.40 [0.29; 0.57]  0.37 [0.26; 0.53]  0.41 [0.29; 0.58]  0.34 [0.24; 0.48] | 1  0.59 [0.42;0.84]  0.53 [0.37;0.75]  0.53 [0.38;0.76]  0.53 [0.38;0.76] | 1  0.69 [0.49;0.99]  0.65 [0.46;0.93]  0.67 [0.47;0.95]  0.65 [0.45;0.93] | 1  0.71 [0.50;1.01]  0.68 [0.47;0.96]  0.70 [0.49;0.99]  0.68 [0.47;0.97] |
| **Individuals with CVRF** | | |  |  |
| Continuous | 0.999 [0.999; 0.999] | 0.999 [0.999;0.999] | 0.999 [0.999;1.00] | 0.999 [0.999;1.00] |
| P for linear trend | <0.001 | 0.02 | 0.07 | 0.10 |
| Quartiles  Inactive  Q1 1-1912  Q2 1913-3690  Q3 3690-7257  Q4 >7527 | 1  0.48 [0.35; 0.65]  0.38 [0.28; 0.52]  0.39 [0.28; 0.53]  0.32 [0.23; 0.44] | 1  0.71 [0.52;0.96]  0.59 [0.43;0.81]  0.54 [0.39;0.73]  0.58 [0.42;0.81] | 1  0.72 [0.53;0.98]  0.63 [0.46;0.86]  0.57 [0.42;0.78]  0.62 [0.44;0.87] | 1  0.73 [0.54;0.996]  0.64 [0.46;0.88]  0.58 [0.42;0.88]  0.64 [0.46;0.90] |
| **Individuals with CVD** |  |  |  |  |
| Continuous | 0.999 [0.999; 0.999] | 0.999 [0.999; 1.00] | 0.999 [0.999; 0.999] | 0.999 [0.999; 1.00] |
| P for linear trend | 0.002 | 0.10 | 0.04 | 0.05 |
| Quartiles  Inactive  Q1 1-1912  Q2 1913-3690  Q3 3690-7257  Q4 >7527 | 1  0.55 [0.35; 0.86]  0.36 [0.21; 0.59]  0.29 [0.17; 0.48]  0.33 [0.19; 0.56] | 1  0.65 [0.41; 1.02]  0.48 [0.29; 0.79]  0.34 [0.20; 0.58]  0.50 [0.28; 0.86] | 1.00  0.66 [0.42; 1.07]  0.53 [0.32; 0.90]  0.38 [0.22; 0.66]  0.60 [0.34; 1.06] | 1.00  0.66 [0.41; 1.06]  0.52 [0.31; 0.87]  0.38 [0.22; 0.65]  0.59 [0.33; 1.05] |
| Model 1 was adjusted for age and sex. *Model 2 was additional adjusted for confounders: income, education, alcohol consumption, smoking behaviour (packyears), nutrient intake (i.e. protein (g/day), fat (g/day), carbohydrate (g/day)), kidney function, arrhythmia, hypothyroid, lung disease, osteoarthritis and rheumatoid arthritis. †Model 3 is further adjusted for mediators: glucose levels, total cholesterol, diastolic blood pressure, systolic blood pressure, BMI, and sleep. | | | | |
