# Supplementary material for: Dose–response association between moderate to vigorous physical activity and incident morbidity and mortality for individuals with a different cardiovascular health status: A cohort study among 142,493 adults from the Netherlands
Source: PLoS Med. 2021 Dec 2;18(12):e1003845. doi: 10.1371/journal.pmed.1003845 (PMC8638933; doi:10.1371/journal.pmed.1003845)
Supplement: S4 Table — (DOCX) [file pmed.1003845.s006.docx]

**S4 Table**. Sensitivity analyses for reverse causation bias and age-restricted (> 50 years) analyses.

| **Total physical activity (MET-min/week)** | **Primary outcome - All-cause mortality and incident MACE** | |
| --- | --- | --- |
|  | Reverse causation bias analyses  Model 2, adjusted for confounders* excluding individuals who experienced an event or were censored within 2 years of follow-up | Age-restricted analyses  Model 2, adjusted for confounders* including individuals who are above 50 years old |
| **Healthy individuals** | N = 111,441; N cases = 1,908 | N = 25,085; N cases = 1,411 |
| Continuous | 0.999 [0.999; 1.00] | 0.999 [0.999; 1.00] |
| P for linear trend | 0.60 | 0.23 |
| Quartiles  Inactive  Q1 1-1912  Q2 1913-3690  Q3 3690-7257  Q4 >7527 | 1.00  0.78 [0.60; 1.01]  0.71 [0.55; 0.93]  0.70 [0.54; 0.92]  0.73 [0.56; 0.95] | 1.00  0.71 [0.54; 0.94]  0.62 [0.47; 0.82]  0.64 [0.48; 0.85]  0.64 [0.48; 0.85] |
| **Individuals with CVRF** | N = 27,418; N cases = 1,650 | N = 17,431; N cases = 1,795 |
| Continuous | 0.999 [0.999; 1.00] | 0.999 [0.999; 1.00] |
| P for linear trend | 0.47 | 0.40 |
| Quartiles  Inactive  Q1 1-1912  Q2 1913-3690  Q3 3690-7257  Q4 >7527 | 1.00  0.73 [0.59; 0.91]  0.71 [0.57; 0.88]  0.68 [0.54; 0.84]  0.69 [0.55; 0.87] | 1.00  0.65 [0.53; 0.79]  0.64 [0.52; 0.78]  0.61 [0.50; 0.75]  0.64 [0.52; 0.80] |
| **Individuals with CVD** | N = 2,053; N cases = 579 | N = 1,932; N cases = 840 |
| Continuous | 0.999 [0.999; 1.00] | 0.999 [0.999; 1.00] |
| P for linear trend | 0.08 | 0.58 |
| Quartiles  Inactive  Q1 1-1912  Q2 1913-3690  Q3 3690-7257  Q4 >7527 | 1.00  0.88 [0.62; 1.25]  0.93 [0.65; 1.33]  0.80 [0.56; 1.15]  0.71 [0.48; 1.04] | 1.00  0.80 [0.61; 1.06]  0.82 [0.62; 1.10]  0.72 [0.54; 0.96]  0.81 [0.60; 1.10] |
| *Model 2 was adjusted for age, sex, income, education, alcohol consumption, smoking behaviour (packyears), nutrient intake (i.e. protein (g/day), fat (g/day), carbohydrate (g/day)), kidney function, arrhythmia, hypothyroid, lung disease, osteoarthritis and rheumatoid arthritis. CVD = cardiovascular disease; CVRF = cardiovascular risk factors; MACE = major adverse cardiovascular events; MET = metabolic equivalent of task | | |
