# Supplementary material for: Dose–response association between moderate to vigorous physical activity and incident morbidity and mortality for individuals with a different cardiovascular health status: A cohort study among 142,493 adults from the Netherlands
Source: PLoS Med. 2021 Dec 2;18(12):e1003845. doi: 10.1371/journal.pmed.1003845 (PMC8638933; doi:10.1371/journal.pmed.1003845)
Supplement: S5 Table — CI, confidence interval; HR, hazard ratio; MACE, major adverse cardiovascular events; MVPA, moderate to vigorous physical activity. (DOCX) [file pmed.1003845.s007.docx]

**S5 Table.** Hazard ratios (95% CI) for the association between leisure moderate to vigorous physical activity and all-cause mortality and MACE.

| **Leisure physical activity**  **(MET-min/week)** | **Primary outcome - All-cause mortality and incident MACE** | | | | | |
| --- | --- | --- | --- | --- | --- | --- |
|  | Unadjusted model | | Model 1, adjusted for age and sex | Model 2, adjusted for confounders* | | Model 3, adjusted for confounders and mediators† |
| **Healthy individuals** | |  | | |  | |
| Continuous | 0.999 [0.999;0.999] | | 0.999 [0.999;0.999] | 0.999 [0.999;1.00] | | 0.999 [0.999;1.00] |
| P for linear trend | <0.001 | | <0.001 | 0.05 | | 0.16 |
| Quartiles  Inactive  Q1 1-1109  Q2 1110-2135  Q3 2136-3731  Q4 >3731 | 1  0.67 [0.57; 0.79], P<0.001  0.63 [0.54; 0.77], P<0.001  0.71 [0.60; 0.83], P<0.001  0.89 [0.76; 1.05], P 0.16 | | 1  0.76 [0.64;0.90], P 0.001  0.67 [0.57;0.79], P<0.001  0.65 [0.55;0.76], P<0.001  0.64 [0.54;0.75], P<0.001 | 1  0.86 [0.73;1.01], P 0.07  0.79 [0.67;0.94], P 0.006  0.78 [0.66;0.92], P 0.003  0.77 [0.66;0.91], P 0.002 | | 1  0.87 [0.74;1.04], P 0.13  0.81 [0.69;0.96], P 0.02  0.80 [0.68;0.95], P 0.009  0.81 [0.69;0.95], P 0.01 |
| **Individuals with CVRF** | |  | | |  | |
| Continuous | 0.999 [0.999;0.999] | | 0.999 [0.999;0.999] | 0.999 [0.999;1.00] | | 0.999 [0.999;1.00] |
| P for linear trend | 0.006 | | 0.003 | 0.06 | | 0.19 |
| Quartiles  Inactive  Q1 1-1109  Q2 1110-2135  Q3 2136-3731  Q4 >3731 | 1  0.66 [0.57; 0.78], P<0.001  0.61 [0.52; 0.72], P<0.001  0.66 [0.56; 0.77], P<0.001  0.79 [0.65; 0.92], P 0.002 | | 1  0.77 [0.66;0.91], P 0.002  0.70 [0.59;0.82], P<0.001  0.67 [0.57;0.78], P<0.001  0.65 [0.56;0.76], P<0.001 | 1  0.82 [0.70;0.96], P 0.01  0.76 [0.65;0.90], P 0.001  0.75 [0.64;0.88], P<0.001  0.73 [0.63;0.85], P<0.001 | | 1  0.84 [0.72;0.99], P 0.03  0.80 [0.68;0.94], P 0.006  0.79 [0.67;0.93], P 0.004  0.77 [0.66;0.90], P 0.001 |
| **Individuals with CVD** | |  | | |  | |
| Continuous | 0.999 [0.999;0.999] | | 0.999 [0.999;0.999] | 0.999 [0.999;1.00] | | 0.999 [0.999;1.00] |
| P for linear trend | 0.001 | | 0.02 | 0.14 | | 0.20 |
| Quartiles  Inactive  Q1 1-1109  Q2 1110-2135  Q3 2136-3731  Q4 >3731 | 1  0.78 [0.62; 0.98], P 0.04  0.83 [0.66; 1.05], P 0.12  0.78 [0.62; 0.97], P 0.03  0.74 [0.60; 0.92], P 0.007 | | 1  0.80 [0.64; 1.00], P 0.05  0.85 [0.68; 1.07], P 0.18  0.77 [0.62; 0.96], P 0.02  0.70 [0.56; 0.87], P 0.001 | 1  0.91 [0.74; 1.15], P 0.43  1.04 [0.70; 1.32], P 0.73  0.94 [0.69; 1.19], P 0.62  0.84 [0.67; 1.05], P 0.13 | | 1  0.89 [0.70; 1.12], P 0.33  1.03 [0.81; 1.31], P 0.79  0.93 [0.74; 1.18], P 0.56  0.84 [0.67; 1.05], P 0.12 |
| Model 1 was adjusted for age and sex. *Model 2 was additional adjusted for confounders: income, education, alcohol consumption, smoking behaviour (packyears), nutrient intake (i.e. protein (g/day), fat (g/day), carbohydrate (g/day)), kidney function, arrhythmia, hypothyroid, lung disease, osteoarthritis, rheumatoid arthritis and non-leisure physical activity. †Model 3 is further adjusted for mediators: glucose levels, total cholesterol, diastolic blood pressure, systolic blood pressure, body mass index, and sleep. CVD = cardiovascular disease; CVRF = cardiovascular risk factors; MACE = major adverse cardiovascular events; MET = metabolic equivalent of task | | | | | | |
