# Supplementary material for: Dose–response association between moderate to vigorous physical activity and incident morbidity and mortality for individuals with a different cardiovascular health status: A cohort study among 142,493 adults from the Netherlands
Source: PLoS Med. 2021 Dec 2;18(12):e1003845. doi: 10.1371/journal.pmed.1003845 (PMC8638933; doi:10.1371/journal.pmed.1003845)
Supplement: S6 Table — CI, confidence interval; HR, hazard ratio; MACE, major adverse cardiovascular events; MVPA, moderate to vigorous physical activity. (DOCX) [file pmed.1003845.s008.docx]

| **S6 Table.** Hazard ratios (95% CI) for the association between leisure moderate to vigorous physical activity and cardiovascular mortality and incident MACE. | | | | |
| --- | --- | --- | --- | --- |
| **Leisure physical activity**  **(MET-min/week)** | **Secondary outcome – CVD mortality and incident MACE** | | | |
|  | Unadjusted model | Model 1, adjusted for age and sex | Model 2, adjusted for confounders* | Model 3, adjusted for confounders and mediators† |
| **Healthy individuals** | | |  |  |
| Continuous | 0.999 [0.999;0.999] | 1.00 [0.999;0.999] | 0.999 [0.999;0.999] | 1.00 [0.999;1.00] |
| P for linear trend | <0.001 | 0.001 | 0.04 | 0.13 |
| Quartiles  Inactive  Q1 1-1109  Q2 1110-2135  Q3 2136-3731  Q4 >3731 | 1  0.66 [0.54; 0.81]  0.66 [0.54; 0.81]  0.71 [0.59; 0.87]  0.90 [0.74; 1.09] | 1  0.75 [0.61;0.91]  0.70 [0.57;0.85]  0.64 [0.53;0.79]  0.63 [0.52;0.77] | 1  0.86 [0.70;1.05]  0.84 [0.69;1.03]  0.79 [0.65;0.97]  0.78 [0.64;0.96] | 1  0.88 [0.72;1.08]  0.87 [0.71;1.06]  0.82 [0.67;1.002]  0.82 [0.67;1.004] |
| **Individuals with CVRF** | | |  |  |
| Continuous | 0.999 [0.999;0.999] | 0.999[0.999;1.00] | 1.00 [0.999;1.00] | 1.00 [0.999;1.00] |
| P for linear trend | <0.001 | 0.25 | 0.89 | 0.64 |
| Quartiles  Inactive  Q1 1-1109  Q2 1110-2135  Q3 2136-3731  Q4 >3731 | 1  0.67 [0.55; 0.80]  0.65 [0.54; 0.78]  0.69 [0.58; 0.83]  0.86 [0.73; 1.03] | 1  0.76 [0.63;0.92]  0.71 [0.60;0.87]  0.69 [0.58;0.83]  0.71 [0.59;0.84] | 1  0.81 [0.68;0.98]  0.80 [0.66;0.96]  0.79 [0.66;0.95]  0.81 [0.68;0.97] | 1  0.83 [0.70;1.01]  0.84 [0.70;1.02]  0.84 [0.69;1.01]  0.86 [0.72;1.04] |
| **Individuals with CVD** | | |  |  |
| Continuous | 0.999 [0.999; 1.00] | 0.999 [0.999;0.999] | 0.999 [0.999;1.00] | 0.999 [0.999;1.00] |
| P for linear trend | 0.08 | 0.02 | 0.15 | 0.22 |
| Quartiles  Inactive  Q1 1-1109  Q2 1110-2135  Q3 2136-3731  Q4 >3731 | 1  0.77 [0.61; 0.98]  0.83 [0.65; 1.05]  0.77 [0.61; 0.98]  0.72 [0.58; 0.91] | 1.00  0.79 [0.62; 0.995]  0.85 [0.67; 1.08]  0.77 [0.61; 0.98]  0.70 [0.56; 0.87] | 1.00  0.90 [0.70; 1.14]  1.04 [0.82; 1.32]  0.95 [0.75; 1.21]  0.84 [0.66; 1.05] | 1.00  0.89 [0.69; 1.12]  1.03 [0.81; 1.34]  0.94 [0.74; 1.20]  0.84 [0.66; 1.06] |
| Model 1 was adjusted for age and sex. *Model 2 was additional adjusted for confounders: income, education, alcohol consumption, smoking behaviour (packyears), nutrient intake (i.e. protein (g/day), fat (g/day), carbohydrate (g/day)), kidney function, arrhythmia, hypothyroid, lung disease, osteoarthritis , rheumatoid arthritis and non-leisure physical activity. †Model 3 is further adjusted for mediators: glucose levels, total cholesterol, diastolic blood pressure, systolic blood pressure, body mass index, and sleep. CVD = cardiovascular disease; CVRF = cardiovascular risk factors; MACE = major adverse cardiovascular events; MET = metabolic equivalent of task | | | | |
