# Supplementary material for: Dose–response association between moderate to vigorous physical activity and incident morbidity and mortality for individuals with a different cardiovascular health status: A cohort study among 142,493 adults from the Netherlands
Source: PLoS Med. 2021 Dec 2;18(12):e1003845. doi: 10.1371/journal.pmed.1003845 (PMC8638933; doi:10.1371/journal.pmed.1003845)
Supplement: S7 Table — CI, confidence interval; HR, hazard ratio; MVPA, moderate to vigorous physical activity. (DOCX) [file pmed.1003845.s009.docx]

| **S7 Table.** Hazard ratios (95% CI) for the association between leisure moderate to vigorous physical activity and all-cause mortality. | | | | |
| --- | --- | --- | --- | --- |
| **Leisure physical activity**  **(MET-min/week)** | **Secondary outcome – All-cause mortality** | | | |
|  | Unadjusted model | Model 1, adjusted for age and sex | Model 2, adjusted for confounders* | Model 3, adjusted for confounders and mediators† |
| **Healthy individuals** |  |  |  |  |
| Continuous | 1.00 [1.00; 1.00] | 0.999 [0.999;0.999] | 0.999 [0.999;1.00] | 0.999 [0.999;1.00] |
| P for linear trend | 0.03 | 0.006 | 0.11 | 0.19 |
| Quartiles  Inactive  Q1 1-1109  Q2 1110-2135  Q3 2136-3731  Q4 >3731 | 1  0.67 [0.53; 0.87]  0.54 [0.41; 0.70]  0.66 [0.51; 0.85]  0.80 [0.62;1.02] | 1  0.77 [0.60;0.99]  0.58 [0.45;0.75]  0.62 [0.48;0.80]  0.58 [0.45;0.75] | 1  0.87 [0.67;1.12]  0.67 [0.52;0.88]  0.74 [0.57;0.96]  0.70 [0.54;0.91] | 1  0.89 [0.69;1.14]  0.69 [0.53;0.90]  0.76 [0.59;0.99]  0.73 [0.56;0.95] |
| **Individuals with CVRF** | |  |  |  |
| Continuous | 0.999 [0.999; 0.999] | 0.999 [0.999;0.999] | 0.999 [0.999;0.999] | 0.999 [0.999;0.999] |
| P for linear trend | <0.001 | <0.001 | <0.001 | <0.001 |
| Quartiles  Inactive  Q1 1-1109  Q2 1110-2135  Q3 2136-3731  Q4 >3731 | 1  0.64 [0.49; 0.82]  0.49 [0.37; 0.65]  0.55 [0.42; 0.72]  0.57 [0.44; 0.75] | 1  0.83 [0.64;1.07]  0.63 [0.48;0.83]  0.62 [0.48;0.82]  0.50 [0.38;0.65] | 1  0.84 [0.65;1.10]  0.68 [0.51;0.90]  0.67 [0.51;0.89]  0.54 [0.41;0.71] | 1  0.85 [0.65;1.10]  0.68 [0.52;0.90]  0.68 [0.52;0.90]  0.54 [0.42;0.72] |
| **Individuals with CVD** | |  |  |  |
| Continuous | 0.999 [0.999; 1.00] | 0.999 [0.999;0.999] | 0.999 [0.999;1.00] | 0.999 [0.999;1.00] |
| P for linear trend | 0.08 | 0.01 | 0.06 | 0.05 |
| Quartiles  Inactive  Q1 1-1109  Q2 1110-2135  Q3 2136-3731  Q4 >3731 | 1  0.69 [0.44; 1.10]  0.63 [0.39; 1.02]  0.55 [0.34; 0.88]  0.50 [0.32; 0.79] | 1.00  0.78 [0.49; 1.24]  0.76 [0.46; 1.23]  0.61 [0.38; 0.99]  0.47 [0.30; 0.75] | 1.00  0.87 [0.55; 1.40]  0.92 [0.56; 1.52]  0.72 [0.44; 1.19]  0.57 [0.35; 0.93] | 1.00  0.86 [0.53; 1.37]  0.89 [0.54; 1.47]  0.70 [0.42; 1.16]  0.55 [0.34; 0.90] |
| Model 1 was adjusted for age and sex. *Model 2 was additional adjusted for confounders: income, education, alcohol consumption, smoking behaviour (packyears), nutrient intake (i.e. protein (g/day), fat (g/day), carbohydrate (g/day)), kidney function, arrhythmia, hypothyroid, lung disease, osteoarthritis , rheumatoid arthritis and non-leisure physical activity. †Model 3 is further adjusted for mediators: glucose levels, total cholesterol, diastolic blood pressure, systolic blood pressure, body mass index, and sleep. CVD = cardiovascular disease; CVRF = cardiovascular risk factors; MACE = major adverse cardiovascular events; MET = metabolic equivalent of task | | | | |
