# Supplementary material for: Dose–response association between moderate to vigorous physical activity and incident morbidity and mortality for individuals with a different cardiovascular health status: A cohort study among 142,493 adults from the Netherlands
Source: PLoS Med. 2021 Dec 2;18(12):e1003845. doi: 10.1371/journal.pmed.1003845 (PMC8638933; doi:10.1371/journal.pmed.1003845)
Supplement: S8 Table — CI, confidence interval; HR, hazard ratio; MACE, major adverse cardiovascular events; MVPA, moderate to vigorous physical activity. (DOCX) [file pmed.1003845.s010.docx]

**S8 Table.** Hazard ratios (95% CI) for the association between non-leisure moderate to vigorous physical activity and all-cause mortality and MACE.

| **Non-leisure physical activity**  **(MET-min/week)** | **Primary outcome - All-cause mortality and incident MACE** | | | | | |
| --- | --- | --- | --- | --- | --- | --- |
|  | Unadjusted model | | Model 1, adjusted for age and sex | Model 2, adjusted for confounders* | | Model 3, adjusted for confounders and mediators† |
| **Healthy individuals** | |  | | |  | |
| Continuous | 0.999 [0.999; 0.999] | | 1.00 [0.999;1.00] | 1.00 [0.999;1.00] | | 1.00 [0.999;1.00] |
| P for linear trend | <0.001 | | 0.19 | 0.82 | | 0.77 |
| Quartiles  Inactive  Q1 1-407  Q2 408-1080  Q3 1080-4088  Q4 >4088 | 1  0.54 [0.48; 0.61], P<0.001  0.47 [0.41; 0.52], P<0.001  0.47 [0.42; 0.53], P<0.001  0.44 [0.39; 0.49], P<0.001 | | 1  0.90 [0.80;1.01], P 0.07  0.87 [0.77;0.99], P 0.03  0.92 [0.82;1.04], P 0.20  0.99 [0.87;1.13], P 0.90 | 1  0.93 [0.83;1.05], P 0.25  0.91 [0.81;1.03] P 0.14  0.96 [0.85;1.09], P 0.54  0.96 [0.84;1.10], P 0.54 | | 1  0.95 [0.85;1.07], P 0.41  0.94 [0.83;1.06], P 0.29  1.00 [0.88;1.13], P 0.99  0.99 [0.87;1.13], P 0.91 |
| **Individuals with CVRF** | |  | | |  | |
| Continuous | 0.999 [0.999; 0.999] | | 1.00 [0.999;1.00] | 1.00 [0.999;1.00] | | 1.00 [1.00 ;1.00] |
| P for linear trend | <0.001 | | 0.09 | 0.16 | | 0.10 |
| Quartiles  Inactive  Q1 1-407  Q2 408-1080  Q3 1080-4088  Q4 >4088 | 1  0.65 [0.57; 0.72], P<0.001  0.54 [0.48; 0.61], P<0.001  0.56 [0.49; 0.63], P<0.001  0.51 [0.45; 0.59], P<0.001 | | 1  0.84 [0.75;0.95], P 0.005  0.81 [0.71;0.91], P<0.001  0.90 [0.79;1.02], P 0.10  1.02 [0.88;1.18], P 0.80 | 1  0.86 [0.77;0.97], P 0.02  0.84 [0.74;0.95], P 0.006  0.91 [0.80;1.04], P 0.18  1.02 [0.89;1.18], P 0.81 | | 1  0.89 [0.79; 1.00], P 0.06  0.88 [0.77; 0.997], 0.045  0.96 [0.84; 1.09], P 0.54  1.06 [0.92; 1.23], P 0.42 |
| **Individuals with CVD** | |  | | |  | |
| Continuous | 0.999 [0.999; 0.999] | | 0.999 [0.999;1.00] | 0.999 [0.999;1.00] | | 1.00 [0.999;1.00] |
| P for linear trend | <0.001 | | 0.23 | 0.13 | | 0.25 |
| Quartiles  Inactive  Q1 1-407  Q2 408-1080  Q3 1080-4088  Q4 >4088 | 1  0.85 [0.72; 1.00], P 0.06  0.62 [0.51; 0.75], P<0.001  0.78 [0.65; 0.94], P 0.009  0.65 [0.52; 0.81], P<0.001 | | 1  0.91 [0.77; 1.07], P 0.26  0.71 [0.58; 0.86], P<0.001  0.88 [0.73; 1.06], P 0.19  0.80 [0.63; 1.02], P 0.07 | 1  0.98 [0.83; 1.15], P 0.78  0.76 [0.62; 0.93], P 0.008  0.94 [0.78; 1.13], P 0.49  0.79 [0.62; 1.01], P 0.06 | | 1  1.01 [0.85; 1.20], P 0.90  0.79 [0.64; 0.97], P 0.02  0.99 [0.82; 1.20], P 0.92  0.84 [0.66; 1.08], P 0.18 |
| Model 1 was adjusted for age and sex. *Model 2 was additional adjusted for confounders: income, education, alcohol consumption, smoking behaviour (packyears), nutrient intake (i.e. protein (g/day), fat (g/day), carbohydrate (g/day)), kidney function, arrhythmia, hypothyroid, lung disease, osteoarthritis and rheumatoid arthritis. †Model 3 is further adjusted for mediators: glucose levels, total cholesterol, diastolic blood pressure, systolic blood pressure, body mass index, and sleep. CVD = cardiovascular disease; CVRF = cardiovascular risk factors; MACE = major adverse cardiovascular events; MET = metabolic equivalent of task | | | | | | |
