# Supplementary material for: Dose–response association between moderate to vigorous physical activity and incident morbidity and mortality for individuals with a different cardiovascular health status: A cohort study among 142,493 adults from the Netherlands
Source: PLoS Med. 2021 Dec 2;18(12):e1003845. doi: 10.1371/journal.pmed.1003845 (PMC8638933; doi:10.1371/journal.pmed.1003845)
Supplement: S9 Table — CI, confidence interval; HR, hazard ratio; MACE, major adverse cardiovascular events; MVPA, moderate to vigorous physical activity. (DOCX) [file pmed.1003845.s011.docx]

| **S9 Table.** Hazard ratios (95% CI) for the association between non-leisure moderate to vigorous physical activity and cardiovascular mortality and incident MACE. | | | | |
| --- | --- | --- | --- | --- |
| **Non-leisure physical activity**  **(MET-min/week)** | **Secondary outcome – CVD mortality and incident MACE** | | | |
|  | Unadjusted model | Model 1, adjusted for age and sex | Model 2, adjusted for confounders* | Model 3, adjusted for confounders and mediators† |
| **Healthy individuals** | | |  |  |
| Continuous | 0.999 [0.999; 0.999] | 1.00 [0.999;1.00] | 0.999 [0.999;1.00] | 1.00 [0.999;1.00] |
| P for linear trend | <0.001 | 0.29 | 0.90 | 0.90 |
| Quartiles  Inactive  Q1 1-407  Q2 408-1080  Q3 1080-4088  Q4 >4088 | 1  0.53 [0.46; 0.61]  0.45 [0.39; 0.52]  0.43 [0.37; 0.49]  0.44 [0.38; 0.51] | 1  0.87 [0.76;1.00]  0.84 [0.73;0.98]  0.84 [0.72;0.97]  0.98 [0.84;1.14] | 1  0.91 [0.79;1.05]  0.88 [0.76;1.03]  0.88 [0.76;1.02]  0.96 [0.82;1.12] | 1  0.92 [0.80;1.07]  0.90 [0.77;1.04]  0.90 [0.77;1.05]  0.97 [0.83;1.13] |
| **Individuals with CVRF** | | |  |  |
| Continuous | 0.999 [0.999; 0.999] | 1.00 [0.999;1.00] | 1.00 [0.999;1.00] | 1.00 [0.999;1.00] |
| P for linear trend | <0.001 | 0.24 | 0.39 | 0.34 |
| Quartiles  Inactive  Q1 1-407  Q2 408-1080  Q3 1080-4088  Q4 >4088 | 1  0.66 [0.58; 0.75]  0.53 [0.46; 0.61]  0.57 [0.49; 0.65]  0.51 [0.44; 0.60] | 1  0.84 [0.74;0.96]  0.76 [0.66;0.88]  0.87 [0.75;1.01]  0.94 [0.80;1.11] | 1  0.86 [0.76;0.99]  0.80 [0.69;0.92]  0.89 [0.77;1.03]  0.94 [0.80;1.11] | 1  0.89 [0.78;1.01]  0.83 [0.71;0.96]  0.92 [0.79;1.07]  0.97 [0.82;1.14] |
| **Individuals with CVD** | | |  |  |
| Continuous | 0.999 [0.999; 0.999] | 0.999 [0.999;1.00] | 0.999 [0.999;1.00] | 0.999 [0.999;1.00] |
| P for linear trend | 0.004 | 0.14 | 0.06 | 0.12 |
| Quartiles  Inactive  Q1 1-407  Q2 408-1080  Q3 1080-4088  Q4 >4088 | 1  0.89 [0.75; 1.06]  0.66 [0.54; 0.80]  0.79 [0.65; 0.96]  0.66 [0.52; 0.84] | 1  0.93 [0.79; 1.11]  0.72 [0.59; 0.89]  0.86 [0.71; 1.05]  0.78 [0.61; 0.998] | 1  1.00 [0.85; 1.20]  0.78 [0.63; 0.96]  0.92 [0.75; 1.12]  0.76 [0.59; 1.01] | 1  1.03 [0.87; 1.24]  0.81 [0.65; 0.99]  0.96 [0.79; 1.18]  0.81 [0.63; 1.04] |
| Model 1 was adjusted for age and sex. *Model 2 was additional adjusted for confounders: income, education, alcohol consumption, smoking behaviour (packyears), nutrient intake (i.e. protein (g/day), fat (g/day), carbohydrate (g/day)), kidney function, arrhythmia, hypothyroid, lung disease, osteoarthritis and rheumatoid arthritis. †Model 3 is further adjusted for mediators: glucose levels, total cholesterol, diastolic blood pressure, systolic blood pressure, body mass index, and sleep. CVD = cardiovascular disease; CVRF = cardiovascular risk factors; MACE = major adverse cardiovascular events; MET = metabolic equivalent of task | | | | |
