# Supplementary material for: Dose–response association between moderate to vigorous physical activity and incident morbidity and mortality for individuals with a different cardiovascular health status: A cohort study among 142,493 adults from the Netherlands
Source: PLoS Med. 2021 Dec 2;18(12):e1003845. doi: 10.1371/journal.pmed.1003845 (PMC8638933; doi:10.1371/journal.pmed.1003845)
Supplement: S10 Table — CI, confidence interval; HR, hazard ratio; MVPA, moderate to vigorous physical activity. (DOCX) [file pmed.1003845.s012.docx]

| **S10 Table.** Hazard ratios (95% CI) for the association between non-leisure moderate to vigorous physical activity and all-cause mortality. | | | | | | |
| --- | --- | --- | --- | --- | --- | --- |
| **Non-leisure physical activity**  **(MET-min/week)** | **Secondary outcome – All-cause mortality** | | | | |  |
|  | Unadjusted model | Model 1, adjusted for age and sex | Model 2, adjusted for confounders* | Model 3, adjusted for confounders and mediators† |  |  |
| **Healthy individuals** |  |  |  |  |  |  |
| Continuous | 0.999 [0.999; 0.999] | 1.00 [0.999;1.00] | 1.00 [0.999;1.00] | 1.00 [0.999;1.00] |  |  |
| P for linear trend | <0.001 | 0.53 | 0.91 | 0.81 |  |  |
| Quartiles  Inactive  Q1 1-407  Q2 408-1080  Q3 1080-4088  Q4 >4088 | 1  0.58 [0.48; 0.70]  0.51 [0.42; 0.61]  0.53 [0.44; 0.64]  0.41 [0.34; 0.51] | 1  0.98 [0.81;1.19]  0.97 [0.79;1.18]  1.07 [0.88;1.31]  1.01 [0.81;1.25] | 1  1.01 [0.84;1.22]  1.00 [0.82;1.22]  1.11 [0.91;1.36]  0.98 [0.79;1.22] | 1  1.04 [0.86;1.26]  1.03 [0.84;1.26]  1.17 [0.96;1.42]  1.01 [0.81;1.26] |  |  |
| **Individuals with CVRF** | |  |  |  |  |  |
| Continuous | 0.999 [0.999; 1.00] | 1.00 [0.999;1.00] | 1.00 [0.999;1.00] | 1.00 [0.999 ;1.00] |  |  |
| P for linear trend | 0.24 | 0.20 | 0.23 | 0.13 |  |  |
| Quartiles  Inactive  Q1 1-407  Q2 408-1080  Q3 1080-4088  Q4 >4088 | 1  0.55 [0.44; 0.68]  0.49 [0.39; 0.61]  0.49 [0.39; 0.61]  0.42 [0.32; 0.54] | 1  0.79 [0.64;0.98]  0.84 [0.67;1.06]  0.97 [0.77;1.23]  1.19 [0.91;1.56] | 1  0.80 [0.65;0.99]  0.87 [0.69;1.09]  0.99 [0.78;1.25]  1.18 [0.90;1.56] | 1  0.84 [0.68;1.05]  0.94 [0.74;1.18]  1.08 [0.86;1.38]  1.27 [0.95;1.68] |  |  |
| **Individuals with CVD** | |  |  |  |  |  |
| Continuous | 0.999 [0.999; 0.999] | 0.999 [0.999;1.00] | 1.00 [0.999;1.00] | 1.00 [0.999;1.00] |  |  |
| P for linear trend | 0.01 | 0.64 | 0.35 | 0.22 |  |  |
| Quartiles  Inactive  Q1 1-407  Q2 408-1080  Q3 1080-4088  Q4 >4088 | 1  0.63 [0.43; 0.92]  0.34 [0.20; 0.57]  0.59 [0.39; 0.91]  0.38 [0.21; 0.70] | 1.00  0.81 [0.55; 1.19]  0.60 [0.35; 1.03]  0.98 [0.64; 1.52]  1.01 [0.54; 1.88] | 1.00  0.81 [0.55; 1.19]  0.62 [0.36; 1.06]  1.06 [0.68; 1.65]  1.18 [0.62; 2.23] | 1.00  0.86 [0.58; 1.27]  0.68 [0.39; 1.17]  1.20 [0.76; 1.87]  1.30 [0.76; 2.47] |  |  |
| Model 1 was adjusted for age and sex. *Model 2 was additional adjusted for confounders: income, education, alcohol consumption, smoking behaviour (packyears), nutrient intake (i.e. protein (g/day), fat (g/day), carbohydrate (g/day)), kidney function, arrhythmia, hypothyroid, lung disease, osteoarthritis and rheumatoid arthritis. †Model 3 is further adjusted for mediators: glucose levels, total cholesterol, diastolic blood pressure, systolic blood pressure, body mass index, and sleep. CVD = cardiovascular disease; CVRF = cardiovascular risk factors; MACE = major adverse cardiovascular events; MET = metabolic equivalent of task | | | | | |  |
