# Supplementary material for: Dose–response association between moderate to vigorous physical activity and incident morbidity and mortality for individuals with a different cardiovascular health status: A cohort study among 142,493 adults from the Netherlands
Source: PLoS Med. 2021 Dec 2;18(12):e1003845. doi: 10.1371/journal.pmed.1003845 (PMC8638933; doi:10.1371/journal.pmed.1003845)
Supplement: S11 Table — CI, confidence interval; HR, hazard ratio; MACE, major adverse cardiovascular events; MVPA, moderate to vigorous physical activity. (DOCX) [file pmed.1003845.s013.docx]

**S11 Table.** Hazard ratios (95% CI) for the association between occupational moderate to vigorous physical activity and all-cause mortality and incident MACE.

| **Occupational**  **physical activity**  **(MET-min/week)** | **Primary outcome - All-cause mortality and incident MACE** | | | | | |
| --- | --- | --- | --- | --- | --- | --- |
|  | Unadjusted model | | Model 1, adjusted for age and sex | Model 2, adjusted for confounders* | | Model 3, adjusted for confounders and mediators† |
| **Healthy individuals** | |  | | |  | |
| Continuous | 0.999 [0.999; 0.999] | | 1.00 [0.999;1.00] | 0.999 [0.999;1.00] | | 1.00 [0.999;1.00] |
| P for linear trend | <0.001 | | 0.34 | 0.81 | | 0.77 |
| Quartiles  Inactive  Q1 1-1949  Q2 1950-4874  Q3 4875-9359  Q4 >9359 | 1  0.82 [0.70; 0.97], P 0.02  0.68 [0.58; 0.80], P<0.001  0.65 [0.55; 0.78], P<0.001  0.72 [0.62; 0.84], P<0.001 | | 1  1.04 [0.89;1.22], P 0.59  1.03 [0.88;1.22], P 0.65  1.03 [0.85;1.23], P 0.77  1.08 [0.92;1.26], P 0.34 | 1  1.05 [0.90;1.24], P 0.53  1.00 [0.85;1.18], P 0.98  0.97 [0.81;1.16], P 0.73  0.998 [0.85;1.17], P 0.98 | | 1  1.05 [0.90;1.24], P 0.53  1.01 [0.86;1.19], P 0.90  0.97 [0.81;1.17], P 0.76  0.99 [0.85;1.16], P 0.92 |
| **Individuals with CVRF** | |  | | |  | |
| Continuous | 0.999 [0.999; 0.999] | | 1.00 [0.999;1.00] | 1.00 [0.999;1.00] | | 1.00 [0.999;1.00] |
| P for linear trend | <0.001 | | 0.13 | 0.24 | | 0.20 |
| Quartiles  Inactive  Q1 1-1949  Q2 1950-4874  Q3 4875-9359  Q4 >9359 | 1  0.79 [0.65; 0.95], P 0.01  0.66 [0.54; 0.81], P<0.001  0.66 [0.53; 0.82], P<0.001  0.65 [0.53; 0.79], P<0.001 | | 1  1.00 [0.83;1.20], P 0.96  1.03 [0.84;1.27], P 0.75  1.10 [0.88;1.36], P 0.40  1.12 [0.91;1.37], P 0.27 | 1  1.01 [0.84;1.22], P 0.88  1.04 [0.85;1.27], P 0.72  1.05 [0.85;1.31], P 0.64  1.10 [0.90;1.36], P 0.34 | | 1  1.04 [0.86;1.25], P 0.71  1.05 [0.86;1.29], P 0.62  1.07 [0.86;1.34], P 0.52  1.11 [0.90;1.36], P 0.32 |
| **Individuals with CVD** | |  | | |  | |
| Continuous | 0.999 [0.999; 0.999] | | 0.999 [0.999;1.00] | 0.999 [0.999;1.00] | | 0.999 [0.999;1.00] |
| P for linear trend | 0.01 | | 0.39 | 0.18 | | 0.28 |
| Quartiles  Inactive  Q1 1-1949  Q2 1950-4874  Q3 4875-9359  Q4 >9359 | 1  0.76 [0.57; 1.01], P 0.06  0.82 [0.61; 1.10], P 0.19  0.69 [0.48; 0.998], P 0.049  0.70 [0.49; 0.998], P 0.049 | | 1  0.82 [0.61; 1.10], P 0.19  0.92 [0.68; 1.25], P 0.59  0.82 [0.57; 1.18], P 0.29  0.86 [0.60; 1.23], P 0.41 | 1  0.82 [0.61; 1.10], P 0.18  0.90 [0.67; 1.22], P 0.51  0.79 [0.54; 1.14], P 0.21  0.79 [0.55; 1.13], P 0.19 | | 1  0.87 [0.65; 1.17], P 0.36  0.98 [0.72; 1.34], P 0.94  0.80 [0.55; 1.16], P 0.25  0.81 [0.56; 1.17], P 0.26 |
| Model 1 was adjusted for age and sex. *Model 2 was additional adjusted for confounders: income, education, alcohol consumption, smoking behaviour (packyears), nutrient intake (i.e. protein (g/day), fat (g/day), carbohydrate (g/day)), kidney function, arrhythmia, hypothyroid, lung disease, osteoarthritis and rheumatoid arthritis. †Model 3 is further adjusted for mediators: glucose levels, total cholesterol, diastolic blood pressure, systolic blood pressure, body mass index, and sleep. CVD = cardiovascular disease; CVRF = cardiovascular risk factors; MACE = major adverse cardiovascular events; MET = metabolic equivalent of task | | | | | | |
