# Supplementary material for: Dose–response association between moderate to vigorous physical activity and incident morbidity and mortality for individuals with a different cardiovascular health status: A cohort study among 142,493 adults from the Netherlands
Source: PLoS Med. 2021 Dec 2;18(12):e1003845. doi: 10.1371/journal.pmed.1003845 (PMC8638933; doi:10.1371/journal.pmed.1003845)
Supplement: S12 Table — CI, confidence interval; HR, hazard ratio; MACE, major adverse cardiovascular events; MVPA, moderate to vigorous physical activity. (DOCX) [file pmed.1003845.s014.docx]

| **S12 Table.** Hazard ratios (95% CI) for the association between occupational moderate to vigorous physical activity and cardiovascular mortality and incident MACE. | | | | |
| --- | --- | --- | --- | --- |
| **Occupational**  **physical activity**  **(MET-min/week)** | **Secondary outcome - CVD mortality and incident MACE** | | | |
|  | Unadjusted model | Model 1, adjusted for age and sex | Model 2, adjusted for confounders* | Model 3, adjusted for confounders and mediators† |
| **Healthy individuals** | | |  |  |
| Continuous | 0.999 [0.999; 0.999] | 1.00 [0.999;1.00] | 1.00 [0.999;1.00] | 0.999 [0.999;1.00] |
| P for linear trend | <0.001 | 0.35 | 0.93 | 0.85 |
| Quartiles  Inactive  Q1 1-1949  Q2 1950-4874  Q3 4875-9359  Q4 >9359 | 1  0.81 [0.66; 0.98]  0.73 [0.60; 0.88]  0.67 [0.54; 0.83]  0.76 [0.64; 0.91] | 1  1.01 [0.83;1.23]  1.10 [0.91;1.33]  1.04 [0.84;1.29]  1.08 [0.91;1.31] | 1  1.02 [0.84;1.24]  1.06 [0.88;1.29]  0.98 [0.79;1.22]  1.01 [0.84;1.21] | 1  1.02 [0.84;1.23]  1.07 [0.88;1.29]  0.99 [0.79;1.23]  0.99 [0.82;1.20] |
| **Individuals with CVRF** | | |  |  |
| Continuous | 0.999 [0.999; 0.999] | 1.00 [0.999;1.00] | 1.00 [0.999;1.00] | 1 [0.999;1.00] |
| P for linear trend | <0.001 | 0.25 | 0.40 | 0.36 |
| Quartiles  Inactive  Q1 1-1949  Q2 1950-4874  Q3 4875-9359  Q4 >9359 | 1  0.85 [0.69; 1.04]  0.62 [0.49; 0.79]  0.70 [0.55; 0.89]  0.71 [0.57; 0.88] | 1  1.03 [0.84;1.27]  0.92 [0.72;1.16]  1.09 [0.86;1.39]  1.12 [0.89;1.40] | 1  1.05 [0.85;1.29]  0.93 [0.73;1.18]  1.05 [0.82;1.33]  1.10 [0.87;1.37] | 1  1.07 [0.87;1.32]  0.94 [0.74;1.19]  1.07 [0.84;1.34]  1.10 [0.88;1.38] |
| **Individuals with CVD** | | |  |  |
| Continuous | 0.999 [0.999; 0.999] | 0.999 [0.999;1.00] | 0.999 [0.999;1.00] | 0.999 [0.999;1.00] |
| P for linear trend | 0.02 | 0.27 | 0.10 | 0.15 |
| Quartiles  Inactive  Q1 1-1949  Q2 1950-4874  Q3 4875-9359  Q4 >9359 | 1  0.75 [0.55; 1.02]  0.81 [0.59; 1.11]  0.68 [0.46; 0.99]  0.72 [0.50; 1.03] | 1  0.80 [0.59; 1.09]  0.89 [0.65; 1.22]  0.77 [0.52; 1.13]  0.84 [0.57; 1.22] | 1  0.80 [0.59 ; 1.09]  0.87 [0.63 ; 1.20]  0.73 [0.50 ; 1.08]  0.76 [0.52 ; 1.10] | 1  0.84 [0.62; 1.15]  0.94 [0.68; 1.30]  0.75 [0.50; 1.10]  0.78 [0.53; 1.13] |
| Model 1 was adjusted for age and sex. *Model 2 was additional adjusted for confounders: income, education, alcohol consumption, smoking behaviour (packyears), nutrient intake (i.e. protein (g/day), fat (g/day), carbohydrate (g/day)), kidney function, arrhythmia, hypothyroid, lung disease, osteoarthritis and rheumatoid arthritis. †Model 3 is further adjusted for mediators: glucose levels, total cholesterol, diastolic blood pressure, systolic blood pressure, body mass index, and sleep. CVD = cardiovascular disease; CVRF = cardiovascular risk factors; MACE = major adverse cardiovascular events; MET = metabolic equivalent of task | | | | |
