# Supplementary material for: Dose–response association between moderate to vigorous physical activity and incident morbidity and mortality for individuals with a different cardiovascular health status: A cohort study among 142,493 adults from the Netherlands
Source: PLoS Med. 2021 Dec 2;18(12):e1003845. doi: 10.1371/journal.pmed.1003845 (PMC8638933; doi:10.1371/journal.pmed.1003845)
Supplement: S13 Table — CI, confidence interval; HR, hazard ratio; MVPA, moderate to vigorous physical activity. (DOCX) [file pmed.1003845.s015.docx]

| **S13 Table.** Hazard ratios (95% CI) for the association between occupational moderate to vigorous physical activity and all-cause mortality. | | | | | | | |
| --- | --- | --- | --- | --- | --- | --- | --- |
| **Occupational**  **physical activity**  **(MET-min/week)** | **Secondary outcome - All-cause mortality** | | | | | | |
|  | Unadjusted model | | Model 1, adjusted for age and sex | | Model 2, adjusted for confounders* | | Model 3, adjusted for confounders and mediators† |
|  | |  | |  | |  | |
| Continuous | 0.999 [0.999; 0.999] | | 1.00 [0.999;1.00] | | 0.999 [0.999;1.00] | | 0.999 [0.999;1.00] |
| P for linear trend | <0.001 | | 0.91 | | 0.62 | | 0.65 |
| Quartiles  Inactive  Q1 1-1949  Q2 1950-4874  Q3 4875-9359  Q4 >9359 | 1  0.83 [0.65; 1.07]  0.58 [0.44; 0.77]  0.61 [0.45; 0.82]  0.63 [0.48; 0.81] | | 1  1.08 [0.84;1.39]  0.93 [0.69;1.23]  1.00 [0.74;1.35]  1.02 [0.78;1.32] | | 1  1.09 [0.84;1.40]  0.90 [0.67;1.19]  0.95 [0.70;1.28]  0.95 [0.73;1.24] | | 1  1.09 [0.85;1.41]  0.91 [0.68;1.21]  0.95 [0.70;1.29]  0.95 [0.73;1.25] |
|  | |  | |  | |  | |
| Continuous | 0.999 [0.999; 0.999] | | 1.00 [0.999;1.00] | | 1.00 [0.999;1.00] | | 1.00 [0.999;1.00] |
| P for linear trend | <0.001 | | 0.60 | | 0.70 | | 0.63 |
| Quartiles  Inactive  Q1 1-1949  Q2 1950-4874  Q3 4875-9359  Q4 >9359 | 1  0.60 [0.41; 0.88]  0.74 [0.52; 1.05]  0.44 [0.28; 0.71]  0.41 [0.27; 0.64] | | 1  0.87 [0.59;1.27]  1.49 [1.04;2.11]  0.97 [0.60;1.56]  1.04 [0.67;1.63] | | 1  0.89 [0.61;1.31]  1.48 [1.04;2.10]  0.92 [0.57;1.49]  1.04 [0.66;1.62] | | 1  0.92 [0.63;1.35]  1.49 [1.04;2.12]  0.94 [0.58;1.52]  1.04 [0.66;1.63] |
|  | |  | |  | |  | |
| Continuous | NA | | NA | | NA | | NA |
| P for linear trend | NA | | NA | | NA | | NA |
| Quartiles  Inactive  Q1 1-1949  Q2 1950-4874  Q3 4875-9359  Q4 >9359 | NA | | NA | | NA | | NA |
| Model 1 was adjusted for age and sex. *Model 2 was additional adjusted for confounders: income, education, alcohol consumption, smoking behaviour (packyears), nutrient intake (i.e. protein (g/day), fat (g/day), carbohydrate (g/day)), kidney function, arrhythmia, hypothyroid, lung disease, osteoarthritis and rheumatoid arthritis. †Model 3 is further adjusted for mediators: glucose levels, total cholesterol, diastolic blood pressure, systolic blood pressure, body mass index, and sleep. CVD = cardiovascular disease; CVRF = cardiovascular risk factors; MACE = major adverse cardiovascular events; MET = metabolic equivalent of task; NA = not applicable because event rates were too low to perform the analyses. | | | | | | | |
